# Supplementary material for: Direct solid-phase synthesis of molecular heterooligonuclear lanthanoid-complexes
Source: Nat Commun. 2020 Mar 12;11:1346. doi: 10.1038/s41467-020-15199-8 (PMC7067767; doi:10.1038/s41467-020-15199-8)
Supplement: Supplementary file 1 — Supplementary Information [file 41467_2020_15199_MOESM1_ESM.pdf]

# Direct Solid-Phase Synthesis of Molecular Heterooligonuclear Lanthanoid Complexes

Elisabeth Kreidt, Wolfgang Leis, Michael Seitz*

Institute of Inorganic Chemistry, University of Tübingen, Auf der Morgenstelle 18, 72076 Tübingen, Germany

Email: michael.seitz@uni-tuebingen.de

# Supplementary Information

## Table of Contents

| | | |
|---|---|---|
| 1. | Supplementary Methods | 2 |
| 1.1. | Materials | 4 |
| 1.2. | HPLC Traces | 14 |
| 1.3. | $^1$H NMR Spectra | 20 |
| 1.4. | Absorption/Luminescence Spectra of **4-Ln** | 26 |
| 1.5. | Absorption/Luminescence Spectra of **Fmoc-Lys(Ln)** and Nanocode **Sm-Tb-Eu** | 30 |
| 1.6. | $^1$H DOSY NMR Studies | 35 |
| | | |
| 2. | Supplementary References | 40 |

# 1. Supplementary Methods

Chemicals were purchased from commercial suppliers and used as received unless stated otherwise. Deuterated solvents/reagents had deuterium contents > 99.5%D. For the preparation of the lanthanoid cryptates, lanthanoid salts with 99.99% purity (REO, with respect to contamination with other lanthanoids) were used. $CH_3CN$ used for the synthesis of the cryptates was HPLC-grade. The resin for SPPS (Fmoc-Gly-TentaGel® R PHB) was purchased from Rapp Polymere GmbH (Tübingen, Germany). Trifluoroacetic acid (TFA) was purchased as HPLC-grade and used as received. Unless stated otherwise, *N,N*-Diisopropylethylamine (DIPEA) was purchased (>98%) and used as received without further purification. Column-chromatography was performed with silica gel 60 (Merck, 0.063-0.200 mm), analytical thin layer chromatography (TLC) was done on silica gel 60 $F_{254}$ plates (Merck, coated on aluminium sheets).

NMR Spectroscopy

NMR spectra were measured at 298 K on Bruker AVII+400 ($^{1}$H: 400 MHz, $^{13}$C: 100.6 MHz, $^{19}$F: 376 MHz), AVII+500 ($^{1}$H: 500 MHz) or Avance III HDX 700 ($^{1}$H: 700 MHz) spectrometers. All chemical shifts are reported in parts per million (ppm). For $^{1}$H and $^{13}$C NMR spectra chemical shifts are reported relative to tetramethylsilane (TMS) and the residual solvent signals have been used as internal reference. Observed multiplicities are specified as: singlet (s), doublet (d), triplet (t), quartet (q), and multiplet (m). Further abbreviations: br = broad.

Mass spectrometry

ESI mass spectrometry was measured using Bruker Daltonics Esquire6000 and Bruker Daltonics Esquire 3000plus. MALDI mass spectrometry was performed using Bruker Autoflex with DHB (2,5-dihydroxybenzoic acid) as matrix. The following abbreviations were used for the description of the results obtained from MALDI-MS: $[DHB\text{-}H]^{-}$: deprotonated DHB ≡ $[C_7H_5O_4]^{-}$; “ox.DHB”: oxidized DHB ≡ $[C_7H_4O_4]$. In high-resolution ESI-MS, the species were typically observed as formate adducts, with formate originating from the formic acid added to the samples.

HPLC

Preparative and analytical HPLC runs were performed on a Knauer Azura HPLC system. For preparative reversed-phase HPLC a Lichrospher RP-18e (250×10mm–10µm) column from Merck with a flow rate of 3 mL min$^{-1}$ was used, for analytical reversed-phase HPLC a Lichrospher RP-18e (125×4mm–5µm) column from Merck with a flow rate of 1 mL min$^{-1}$ was used. UV/vis absorption (300 nm) was used for the detection of eluted substances. All samples were filtered with a 0.45 µm nylon membrane filter (GE Healthcare Life Sciences) before

injection. Using mobile phases 1 (degassed HPLC-grade $H_2O$ + 1 vol.-% HPLC-grade $CF_3COOH$) and 2 (degassed HPLC-grade $CH_3CN$), the programs detailed in Supplementary Table 1 were applied:

**Supplementary Table 1.** HPLC gradients with mobile phases 1 and 2 (see text) used for purification of lanthanoid complexes.

HPLC Program **A**

| time [min] | %1 | %2 |
|---|---|---|
| 0 | 85 | 15 |
| 5 | 85 | 15 |
| 19 | 45 | 55 |
| 25 | 45 | 55 |
| 40 | 85 | 15 |
| 50 | 85 | 15 |

HPLC Program **B**

| time [min] | %1 | %2 |
|---|---|---|
| 0 | 85 | 15 |
| 5 | 85 | 15 |
| 19 | 45 | 55 |
| 41 | 0 | 100 |
| 48 | 0 | 100 |
| 78 | 85 | 15 |
| 90 | 85 | 15 |

Photophysical measurements

UV/vis absorption spectra were measured on a Jasco V-770 spectrophotometer using quartz cuvettes (Suprasil, 1 cm pathlength) at room temperature. Steady state emission spectra were acquired on a Horiba Fluorolog-3 DF spectrofluorimeter using quartz cuvettes (Suprasil, 1 cm pathlength) at room temperature. The excitation light source was a 450 W xenon lamp. Emission was monitored at 90° using a Hamamatsu R2658P PMT (UV/vis/NIR, 300 nm < $\lambda_{em}$ < 1010 nm). Spectral selection was achieved by double grating monochromators (excitation: 1200 grooves/nm, blazed at 300 nm, emission: 1200 grooves/nm, blazed at 500 nm).

## 1.1 Materials

Isothiocyanate (*S*)-**S2**

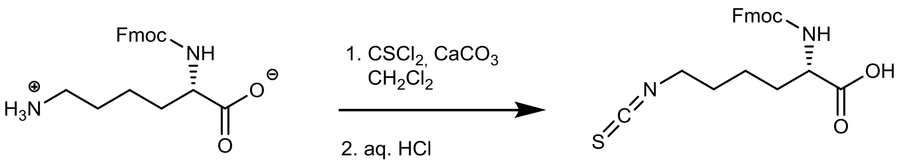


$N_\alpha$-Fmoc-L-lysine (**S1**)

(*S*)-**S2**

**CAUTION:** Thiophosgene is highly toxic (especially after but not limited to skin resorption) and should be handled with appropriate safety measures in place, for the reaction itself and during chromatographic purification!

Anhydrous $CaCO_3$ (29.8 mg, 298 µmol, 1.1 equivs.) was suspended in $CH_2Cl_2$ (200 mL) and stirred vigorously for 30 minutes. A 0.1 M solution of thiophosgene (4.07 mL solution, 46.5 mg, 407 µmol, 1.5 equivs.) in $CH_3CN$ (peptide grade) was added, immediately followed by the Fmoc-protected L-lysine derivative **S1** (100 mg, 271 µmol, 1.0 equiv.). The mixture was stirred for 24 h at room temperature. $H_2O$ (80 mL) was added, followed by 0.1 M aqueous HCl (3.25 mL, 325 µmol, 1.2 equivs.). After stirring vigorously for 90 minutes, the phases were separated, and the neutral aqueous phase was extracted with additional $CH_2Cl_2$ (4 × 100 mL). The combined organic phases were dried ($MgSO_4$) and evaporated to dryness to give a slightly yellow, glassy solid. The crude product was subjected to column chromatography ($SiO_2$, $CH_2Cl_2$/$CH_3OH$, gradient: 25:1 → 9:1, UV detection). TLCs of early fractions containing the product revealed a contamination with another compound which could not be detected using $^1$H NMR or ESI-MS (pos. mode). The substance collected from these fractions was subjected to column-chromatography for a second time ($SiO_2$, $CH_2Cl_2$/$CH_3OH$, gradient: 50:1 → 9:1, UV detection) to give another portion of the pure product. The combined product fractions were dried *in vacuo* overnight to remove traces of solvents. The title compound was obtained as a slightly yellow solid. Total yield: 52.3 mg (47%).

MS (ESI, pos. mode): *m/z* (%) = 413.3 (10), 433.2 (100, $[M+Na]^+$), 449.1 (43, $[M+K]^+$). $^1$H NMR (400 MHz, $CD_2Cl_2$): $\delta$ = 7.79 (d, *J* = 7.6 Hz, 2 H), 7.62 (d, *J* = 7.5 Hz, 2 H), 7.46-7.28 (m, 4 H), 4.49-4.31 (m, 3 H), 4.24 (t, *J* = 6.6 Hz, 1 H), 3.59-3.47 (m, 2 H), 2.00-1.30 (m, 6 H) ppm. $^{13}$C NMR (101 MHz, $CD_2Cl_2$): $\delta$ = 177.1, 157.0, 144.3, 141.8, 130.3, 128.3, 127.6, 125.6, 120.5,

67.6, 47.7, 45.4, 31.9, 30.3, 29.9, 23.1 ppm. TLC: $R_f$ = 0.20 ($SiO_2$, $CH_2Cl_2/CH_3OH$ 15:1, UV detection).

Note: The title compound is stable in $CH_2Cl_2$ solution at room temperature or neat in the solid state at -25°C for several weeks without any apparent decomposition (as monitored by $^1H$ NMR). Yet results of the subsequent reaction with the amino-functionalised cryptates (see below) were found to proceed more efficiently with freshly prepared (*S*)-**S2**.

Synthesis of the Monomers **Fmoc-Lys(Ln)** (via HPLC-purified amino-functionalised lanthanoid cryptates **S4-Ln**):

Lanthanoid Cryptates **S4-Ln**:

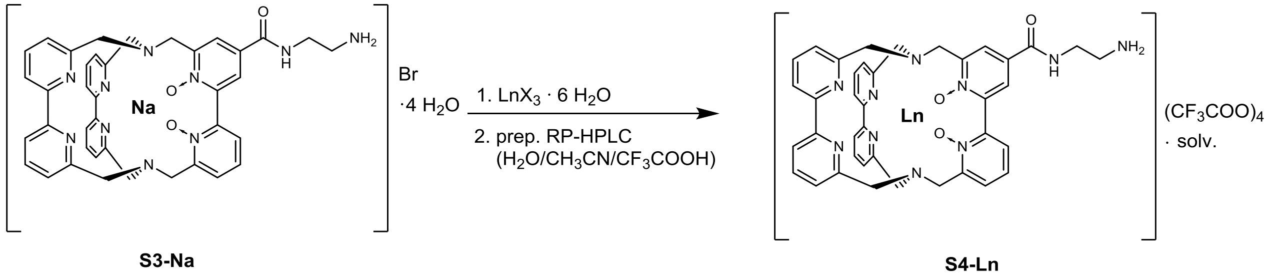


The general procedure for the preparation of the sodium cryptate **S3-Na** has already been described elsewhere.[1]

General Procedure 1 (GP1): Sodium cryptate **S3-Na** (1.0 equiv.) and $LnCl_3 \cdot 6\ H_2O$ (1.7 equivs.) were suspended in $CH_3CN$ (HPLC-grade) and heated to reflux for at least 40 h. After cooling to room temperature, volatile components of the mixture were removed *in vacuo*. The obtained residue was dissolved in $CH_3CN/H_2O$ (1:1, v/v) (about 1.0 mL per 5 mg of **S3-Na**), filtered, and subjected to semi-preparative reversed-phase HPLC using HPLC program **A** (see above). The product was isolated after evaporation to dryness *in vacuo* at room temperature as off-white or faintly yellow solid.

**S4-Sm**:

Following GP1 (see above) starting from **S3-Na** (10.0 mg, 13.0 μmol, 1.0 equiv.), $SmCl_3 \cdot 6\ H_2O$ (8.1 mg, 22 μmol, 1.7 equivs.) in 15 mL $CH_3CN$ (65 h reflux). Yield: 7.6 mg, 5.4 μmol, 42%.

$^{1}H$ NMR (400 MHz, $CD_3OD$): $\delta$ = 9.47 (d, *J* = 7.9 Hz, 1 H), 9.36 (d, *J* = 8.1 Hz, 1 H), 9.03 (d, *J* = 8.0 Hz, 1 H), 8.98-8.88 (m, 2 H), 8.84 (t, *J* = 8.0 Hz, 1 H), 8.46 (d, *J* = 2.4 Hz, 1 H), 8.24-7.95 (m, 6 H), 7.92 (d, *J* = 7.8 Hz, 1 H), 9.47 (d, *J* = 7.9 Hz, 1 H), 7.75 (dd, *J* = 7.8, 2.0 Hz, 1 H), 7.01 (d, *J* = 12.6 Hz, 1 H), 6.73 (d, *J* = 12.6 Hz, 1 H), 6.66-6.58 (m, 2 H), 3.66 (t, *J* = 5.9 Hz, 2 H), 3.15 (t, *J* = 6.0 Hz, 2 H), 3.01-2.89 (m, 2 H), 2.60-2.50 (m, 2 H), 1.49-1.35 (m, 2 H), 0.95-0.77 (m, 2 H), 0.61 (d, *J* = 15.1 Hz, 1 H), 0.50 (d, *J* = 15.3 Hz, 1 H) ppm (see Supplementary Figure 8). $^{19}F$ NMR (376 MHz, $CD_3OD$): $\delta$ = –77.4 (s) ppm. MALDI-MS (matrix: DHB, RP mode, pos. mode): *m/z* (%) = 1118.21 (100, $[M–2O+2DHB–2H]^+$, Sm-isotope pattern). High resolution ESI-MS (pos. mode, formic acid added): $[C_{39}H_{36}N_{10}O_3Sm+HCOO]^{2+}$: calculated: *m/z* = 444.60674, found: *m/z* = 444.60658. HPLC: $t_r$ = 11.8 min (analytical reversed-phase HPLC, HPLC program **A**, see Supplementary Figure 2).

**S4-Eu**:

Following GP1 (see above) starting from **S3-Na** (35.0 mg, 44.0 μmol, 1.0 equiv.), $EuCl_3 \cdot 6\ H_2O$ (27.4 mg, 74.8 μmol 1.7 eq.) in 17 mL $CH_3CN$ (70 h reflux). Yield: 14.3 mg, 10.2 μmol, 23%.

$^{1}H$ NMR (400 MHz, $CD_3OD$): $\delta$ = 27.04 (br s, 1 H), 26.15 (br s, 1 H), 23.92 (br s, 2 H), 13.31 (s, 2 H), 10.39-10.04 (m, 2 H), 9.98-9.88 (m, 1 H), 9.88-9.77 (m, 2 H), 9.29 (t, *J* = 7.8 Hz, 1 H), 9.12 (d, *J* = 7.8 Hz, 1 H), 7.15 (s, 1 H), 7.05-6.75 (m, 3 H), 5.48-5.40 (m, 1 H), 5.10-5.02 (m, 1 H), 4.28-4.19 (m, 1 H), 4.18-4.09 (m, 1 H), 3.93-3.76 (m, 3 H), 3.69-3.61 (m, 1 H), 3.39 (t, *J* = 6.0 Hz, 2 H), 1.76-1.66 (m, 1 H), 1.17-1.10 (m, 1 H), -8.79 to -8.99 (m, 1 H), -9.51 to -9.70 (m, 1 H), -11.19 (br s, 1 H), -12.82 (br s, 1 H) ppm (see Supplementary Figure 9). $^{19}F$ NMR (376 MHz, $CD_3OD$): $\delta$ = -77.5 (s) ppm. MALDI-MS (matrix: DHB, RP mode, pos. mode): *m/z* (%) = 966.15 (100, $[M\ –2O–H+CF_3OO+CH_3CN]^+$, Eu-isotope pattern), 1119.16 (6, $[M–2O+CF_3OO+ox.DHB +CH_3CN+e]^+$). High resolution ESI-MS (pos. mode, formic acid added): $[C_{39}H_{36}EuN_{10}O_3+HCOO]^{2+}$: calculated: *m/z* = 445.10749, found: *m/z* = 445.10750. HPLC: $t_r$ = 11.7 min (analytical reversed-phase HPLC, HPLC program **A**, see Supplementary Figure 3).

**S4-Tb**:

Following GP1 (see above) starting from **S3-Na** (30.0 mg, 37.7 µmol, 1.0 equiv.), $TbCl_3 \cdot 6\ H_2O$ (23.9 mg, 64.1 µmol, 1.7 equivs.) in 17 mL $CH_3CN$ (40 h reflux). Yield: 14.6 mg, 10.3 µmol, 27%.

$^1H$ NMR (500 MHz, $CD_3OD$): $\delta$ = 228.45, 186.01, 86.05, 70.51, 63.09, 51.93, 43.10, 37.51, 33.04, 24.90, 9.13, -14.38, -19.22, -21.77, -22.44, -59.14, -72.76, -95.46, -95.83, -102.88, -110.60, -211.95, -212.47, -273.68, -277.62, -307.42, -328.51 ppm (see Supplementary Figure 10; Note: In the middle region of the spectrum not all signals could be identified unambiguously). $^{19}F$ NMR (376 MHz, $CD_3OD$): $\delta$ = -77.0 (s) ppm. MALDI-MS (matrix: DHB, RP mode, pos. mode): *m/z* (%) = 1125.23 (100, $[M{-}2O{+}\ 2DHB{-}2H]^+$), 1141.24 (4, $[M{-}O{+}2DHB{-}2H]^+$). HR ESI-MS (pos. mode, formic acid added): $[C_{39}H_{36}N_{10}O_3Tb{+}HCOO]^{2+}$: calculated: *m/z* = 448.10954, found: *m/z* = 448.10949. HPLC: $t_r$ = 11.4 min (analytical reversed-phase HPLC, HPLC program **A**, see Supplementary Figure 4).

Lanthanoid Cryptate Monomers **Fmoc-Lys(Ln)**:

General procedure 2 (GP2):

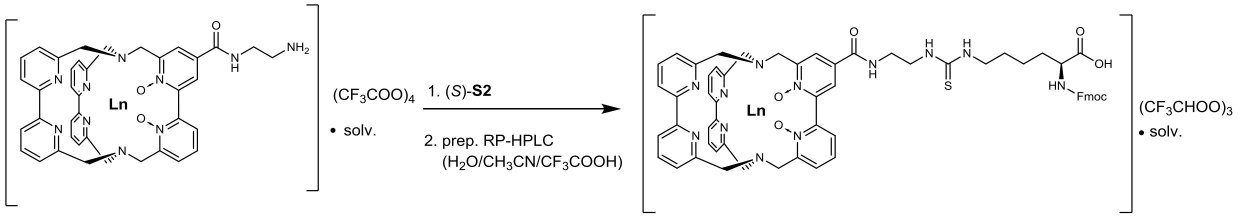


**S4-Ln** **Fmoc-Lys(Ln)**

The racemic lanthanoid cryptate **S4-Ln** was dissolved in MeOH and DIPEA (0.1 M in MeOH, varying amounts > 3 equivalents, see exact amounts below) were added. The mixture was stirred for a few minutes before a solution of (*S*)-**S2** (varying amounts > 1.45 equivalents, see below) in $CH_3OH/CH_2Cl_2$ (1:1, v/v) was added dropwise. After 30 minutes another portion of DIPEA (0.1 M in MeOH, see amounts below) was added, followed by another portion of of (*S*)-**S2** (see amounts below), dissolved in $CH_3OH/CH_2Cl_2$ (1:1, v/v). Stirring was continued at room temperature for a total of 19 hours, volatiles were removed under reduced pressure, and the crude product was dried thoroughly. The material was dissolved in a mixture of $CH_3CN/H_2O/CF_3COOH$ (1:1:0.005, v/v/v) (about 1 mL per 1.5 mg starting **S4-Ln**), filtered, and subjected to semi-preparative reversed-phase HPLC using HPLC program **B** (see above). The

title compounds (off-white or faintly yellow solid) were isolated as a mixture of diastereomers for each lanthanoid after evaporation of the pure fractions to dryness *in vacuo* at room temperature.

**Fmoc-Lys(Sm):**

Following GP2 starting from **S4-Sm** (2.9 mg, 2.1 µmol, 1.0 equiv.) dissolved in $CH_3OH$ (0.5 mL). First addition: DIPEA solution (62 µL, 6.2 µmol, 3.0 equivs.) and (*S*)-**S2** (1.3 mg, 3.1 µmol, 1.5 equivs.) dissolved in $CH_3OH/CH_2Cl_2$ (0.3 mL). Second addition: DIPEA solution (31 µL, 3.1 µmol, 1.5 equivs.) and (*S*)-**S2** (1.3 mg 3.1 µmol, 1.5 equivs.) dissolved in $CH_3OH/CH_2Cl_2$ (0.3 mL). Yield: 0.9 mg, 0.5 µmol, 24%.

$^1H$ NMR (400 MHz, $CD_3OD$): see Supplementary Figure 11 for the mixture of diastereomers. $^{19}F$ NMR (376 MHz, $CD_3OD$): $\delta$ = -77.4 (s) ppm. MALDI-MS (matrix: DHB, RP mode, pos. mode): *m/z* (%) = 1376.27 (100, $[M–2·O–H+DHB–H]^+$, Sm-isotope pattern). High-resolution ESI-MS (pos. mode, formic acid added): $[C_{61}H_{57}N_{12}O_7S_1Sm_1–H+HCOO]^+$: calculated: *m/z* = 1298.33623, found: *m/z* = 1298.33181. HPLC (analytical reversed-phase HPLC, HPLC program A): $t_r$ = 17.1 min (see see Supplementary Figure 5).

**Fmoc-Lys(Eu):**

Following GP2 starting from **S4-Eu** (7.8 mg, 5.6 µmol, 1.0 equiv.), dissolved in $CH_3OH$ (0.5 mL). First addition: DIPEA solution (164 µL, 16.4 µmol, 3 equivs.) and (*S*)-**S2** (3.3 mg, 8.0 µmol, 1.45 equivs.) dissolved in $CH_3OH/CH_2Cl_2$ (1.5 mL). Second addition: DIPEA solution (95 µL, 9.5 µmol, 1.7 equivs.) and (*S*)-**2** (3.9 mg, 9.5 µmol, 1.7 equivs.) dissolved in $CH_3OH/CH_2Cl_2$ (in 1.5 mL). Yield: 2.1 mg, 1.3 µmol, 23%.

$^1H$ NMR (400 MHz, $CD_3OD$): see Supplementary Figure 12 for the mixture of diastereomers. $^{19}F$ NMR (376 MHz, $CD_3OD$): $\delta$ = -77.3 (s) ppm. MALDI-MS (matrix: DHB, RP mode, pos. mode): *m/z* (%) = 1206.36 (26, $[M–3·O–H+e]^+$, Eu isotope pattern), 1222.35 (100, $[M–2·O–H+e]^+$, Eu-isotope pattern), 1375.40 (9, $[M–2·O–H+DHB–H]^+$, Eu-isotope pattern). High resolution ESI-MS (pos. mode, formic acid added): $[C_{61}H_{57}Eu_1N_{12}O_6S_1–H+HCOO]^+$: calculated: *m/z* = 1283.34281, found: *m/z* = 1283.3398. HPLC (analytical reversed-phase HPLC, HPLC program A): $t_r$ = 17.1 min (see see Supplementary Figure 6).

**Fmoc-Lys(Tb):**

Following GP2 starting from **S4-Tb** (3.6 mg, 2.5 μmol, 1.0 equiv.) dissolved in $CH_3OH$ (0.5 mL). First addition: DIPEA solution (76 μL, 7.6 μmol, 3.0 equivs.) and (*S*)-**S2** (1.6 mg, 3.8 μmol, 1.5 equivs.) dissolved in $CH_3OH/CH_2Cl_2$ (0.3 mL). Second addition: DIPEA solution (38 μL, 3.8 μmol, 1.5 equivs.) and (*S*)-**S2** (1.6 mg, 3.8 μmol, 1.5 equivs.) dissolved in $CH_3OH/CH_2Cl_2$ (1.5 mL). Yield: 1.7 mg, 1.0 μmol, 40%.

$^1$H NMR (400 MHz, $CD_3OD$): see Supplementary Figure 13 for the mixture of diastereomers. $^{19}$F NMR (376 MHz, $CD_3OD$): $\delta$ = -77.2 (s) ppm. MALDI-MS (matrix: DHB, RP mode, pos. mode): *m/z* (%) = 1349.33 (45, $[M–4·O–H+DHB–H]^+$), 1365.32 (30, $[M–3·O–H+DHB–H]^+$), 1381.31 (100, $[M–2·O–H+DHB–H]^+$). High resolution ESI-MS (pos. mode, formic acid added): $[C_{61}H_{58}N_{12}O_7S_1Tb_1+HCOO]^{2+}$: calculated: *m/z* = 653.17456, found: *m/z* = 653.17430. HPLC (analytical reversed-phase HPLC, HPLC program A): $t_r$ = 17.1 min (see see Supplementary Figure 7).

Synthesis of the Monomers **Fmoc-Lys(Ln)** (direct synthesis without isolation of cryptates **S4-Ln**):

Note: The lanthanoid cryptate monomers **Fmoc-Lys(Ln)** can also be prepared from the crude complexes **S4-Ln**. In this case, after the synthesis of **S4-Ln** the crude material obtained after evaporation of the solvents is subjected to the next reaction step without any purification. The content of the lanthanoid cryptate in these crude materials can only be estimated. The material was assumed to be the pure chloride ($C_{39}H_{36}N_{10}O_3LnCl_3$) and it is not reasonable to determine yields from these reactions. But as the reaction also proceeds properly with the crude starting material and the reduction of HPLC purifications saves a significant amount of time, this variation is reasonable when larger amounts of lysine-functionalized lanthanoid cryptates **Fmoc-Lys(Ln)** are to be prepared.

General Procedure 3 (GP3):

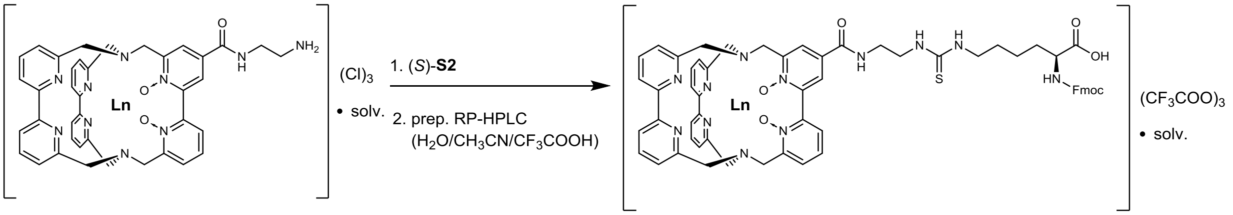


The crude lanthanoid cryptate **S4-Ln** (1.0 equiv.) was dissolved in $CH_3OH$ (0.1 mL per mg starting material) and stirred for a few minutes. DIPEA (0.1 M in $CH_3OH$, 2.0 equivs.) was added and the resulting solution was stirred for a few minutes. (*S*)-**S2** (1.5 equivs.) was dissolved in a 0.1 M solution of DIPEA in $CH_3OH$ (35 µL solution per µmol **S4-Ln**, 3.5 equivs.), the resulting solution was added dropwise to the starting material, upon which a yellow solid precipitated. The mixture was diluted with a freshly prepared mixture of of $CH_3OH/CH_2Cl_2$ (1:1, v/v, about 0.02 mL per mg **S4-Ln**). After 30 minutes, another portion of (S)-**S2** (1.5 equivs) was added to the reaction mixture with about 0.6 mL $CH_3OH/CH_2Cl_2$ (1:1, v/v) per mg **S4-Ln**. After stirring at room temperature for at least 13.5 h. The volatiles were removed and the crude product was dried thoroughly *in vacuo*. The material was dissolved in a mixture of $CH_3CN/H_2O/CF_3COOH$ (1:1:0.005, v/v/v) (about 1mL per 3 mg starting **S4-Ln**), filtered, and subjected to semi-preparative reversed-phase HPLC using HPLC program **B** (see above). When crude **S4-Ln** is used for the preparation of **Fmoc-Lys(Ln)**, unreacted **S3-Na** is transformed into poorly soluble **Fmoc-Lys(Na)** which precipitates from the reaction mixture during the first addition of (*S*)-**S2**. In this case, special care has to be taken to achieve maximum dissolution of the product (note: The use of an ultrasonic bath can help to suspend the poorly soluble and unwanted **Fmoc-Lys(Na)** and to release entrapped **Fmoc-Lys(Ln)**). The title compounds (off-white or faintly yellow solids) were isolated as a mixture of diastereomers for each lanthanoid after evaporation of the pure fractions to dryness *in vacuo* at room temperature.

**Fmoc-Lys(Ln)** prepared by GP3 showed analytical data identical to the materials obtained by the route via GP1 and GP2 (vide supra).

## Peptide Synthesis

Synthesis of the nanocode **Sm-Tb-Eu** was performed via Solid Phase Peptide Synthesis (SPPS) following the Fmoc strategy and using standard techniques.[2] DMF (Acros Organics, 99.8%, for peptide synthesis), piperidine (Roth, 99.5%, for peptide synthesis) and $Ac_2O$ (Merck, EMSURE®, for analysis) were stored under Ar and all mixtures were freshly prepared shortly before use. As solid support Fmoc-Gly-TentaGel® R PHB resin (particle size 90 µm, capacity

0.15-0.20 mmol $g^{-1}$, Wang-type linker) was used. Isotopically enriched Fmoc-protected glycines (Cambridge Isotope Laboratories, Inc.) had chemical purities 98% and were 99% (2-$^{13}C$) or 98% ($^{15}N$) enriched. As coupling reagent HATU was used, as base dry DIPEA (freshly distilled from KOH) was added. The reaction was performed in a custom-build glass-vessel (see Supplementary Figure 1). To minimise adhesion of the resin to the glass surface of the vessel, it was silanized with trimethylsilyl chloride prior to first usage.

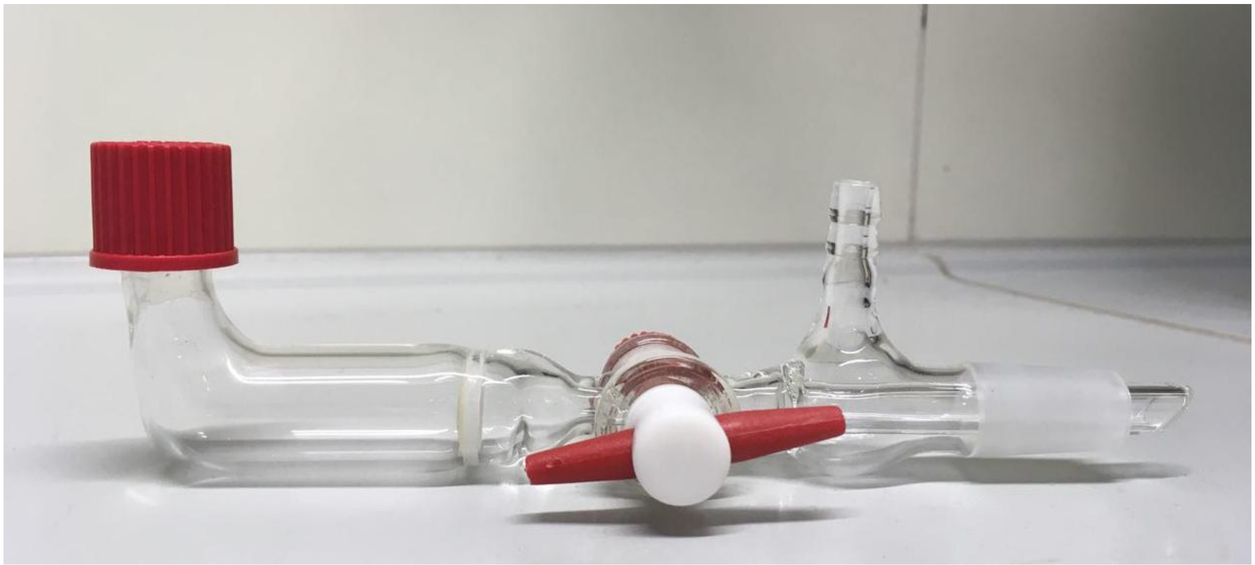

**Supplementary Figure 1.** Custom-build glass-vessel for SPPS.

All equivalents were calculated with respect to a capacity of the resin of 0.20 mmol $g^{-1}$. The following standard procedures were carried out:

• Washing: About three bed volumes (at least 1 mL) of the solvent were added to the reaction vessel, the suspension was shaken for about one minute before the solvent was drained. The procedure was performed a total of five times.
• Swelling of the resin: Prior to the first coupling step, the dry resin was placed in the reaction vessel and at least a three bed volumes of $CH_2Cl_2$ were added. The suspension was shaken carefully and left standing for about 30 minutes before the suspension was shaken for about 20 minutes. The $CH_2Cl_2$ was drained and the resin was washed with DMF (see Washing above).
• Coupling: If not stated otherwise, in the case of commercially available amino acids the Fmoc-protected amino acid (5 equivs.) and HATU (4.9 equivs.) were dissolved in a minimum amount of DMF. After addition of DIPEA (10 equivs.) the mixture was added to the deprotected, washed

resin immediately. The suspension was shaken for at least 30 minutes before the solution was drained and the resin was washed with DMF (see Washing above). In the case of the lanthanoid-containing amino acids the equivalents were reduced and the reaction times were elongated (see below).

• Capping: A 50-fold excess of acetic anhydride/DMF/DIPEA (1:20:1 v/v/v, freshly prepared) was added to the washed resin and shaken for 30 minutes. The solution was drained and the resin was washed with DMF (see Washing above).

• Deprotection: About three bed volumes (at least 1 mL) of 20% piperidine in DMF (v/v) were added to the washed resin. The suspension was gently shaken for about 2 minutes before the solution was drained and the process was repeated two more times. Afterwards the resin was washed with DMF (see Washing above).

• Shrinkage of the resin: The DMF-washed resin was washed with $CH_2Cl_2$ and subsequently with $Et_2O$ (see Washing above). To remove remaining solvent the resin was dried *in vacuo* for several hours and subsequently dried in air overnight.

• Cleavage of the peptide from the resin: About three bed volumes (at least 1 mL) of trifluoroacetic acid (TFA, HPLC-grade) with 5 vol.-% $H_2O$ were added to the shrunk resin and the suspension was shaken for ca. 60 minutes. The solution was drained into a round-bottomed flask equipped with a magnetic stirrer. Typically this step was repeated 1-3 times.

<u>Nanocode Synthesis</u>

Fmoc-Gly-Tentagel® PHB R resin (6.01 mg, equals 1.20 µmol of the bound Fmoc-Gly, 1.0 equiv.) was swelled in the custom-build glass-vessel (see Supplementary Figure 1). After initial Fmoc deprotection, the following steps were performed:

1.) Coupling of the lysine-functionalized samarium cryptate **Fmoc-Lys(Sm)**

**Fmoc-Lys(Sm)** (3.00 mg, 1.80 µmol, 1.5 equivs.), HATU (0.66 mg, 1.74 µmol, 1.45 equivs.), and a solution of DIPEA in DMF (*c* = 0.36 M, 10 µL solution, 3.60 µmol DIPEA, 3.0 equivs.). Total volume of 2.0 mL of DMF, reaction time 16 h. After this coupling step, a capping step and a subsequent deprotection step were performed.

2.) Coupling of the $^{15}N$-labeled glycine Fmoc-Gly($^{15}N$)-OH

Fmoc-Gly($^{15}N$)-OH (1.79 mg, 6.01 µmol, 5.0 equivs.), HATU (2.24 mg, 5.89 µmol, 4.9 equivs.), and a solution of DIPEA in DMF (*c* = 1.20 M, 10 µL solution, 12.0 µmol DIPEA, 10 equivs.). Total volume of 2 mL of DMF, reaction time 11 h. After the solution was drained, the resin was washed and a fresh analogously prepared coupling mixture was added. Total volume of 2 mL of

DMF, reaction time 30 minutes. The coupling procedures was followed by a capping and a deprotection step.

3.) Coupling of the lysine-functionalized terbium cryptate **Fmoc-Lys(Tb)**

**Fmoc-Lys(Tb)** (3.02 mg, 1.80 µmol, 1.5 equivs.), HATU (0.66 mg, 1.74 µmol, 1.45 equivs.), and a solution of DIPEA in DMF ($c$ = 0.36 M, 10 µL solution, 3.60 µmol DIPEA, 3.0 equivs.). Total volume of 2 mL of DMF, reaction time 12 h. After this coupling step, a capping step and a subsequent deprotection step were performed.

4.) Coupling of the 2-$^{13}$C-labeled glycine Fmoc-Gly($^{13}$C)-OH

Fmoc-Gly($^{13}$C)-OH (1.79 mg, 6.01 µmol, 5.0 equivs.), HATU (2.24 mg, 5.89 µmol, 4.9 equivs.), and a solution of DIPEA in DMF ($c$ = 1.20 M, 10 µL solution, 12.0 µmol DIPEA, 10 equivs.). Total volume of 2 mL of DMF, reaction time 9 h. After the solution was drained, the resin was washed and a fresh analogously prepared coupling mixture was added. Total volume of 2 mL of DMF, reaction time 30 minutes. The coupling procedure was followed by a capping and a deprotection step.

5.) Coupling of the lysine-functionalized europium cryptate Fmoc-Lys(Eu)

**Fmoc-Lys(Eu)** (3.00 mg, 1.80 µmol, 1.5 equivs.), HATU (0.66 mg, 1.74 µmol, 1.45 equivs.), and a solution of DIPEA in DMF ($c$ = 0.36 M, 10 µL solution, 3.60 µmol DIPEA, 3.0 equivs.). Total volume of 2 mL of DMF, reaction time 12 h. After this coupling step, a capping step and a subsequent deprotection step were performed.

At the end, the resin was shrunk and treated with three portions of TFA (1 mL each with 5 vol% $H_2O$, 60 minutes reaction time for each addition). The TFA solutions were drained into a round-bottomed flask and evaporated to dryness. Subsequently, $CH_3OH$ (1 mL) was added to the resin. The suspension was shaken for 1 h, the solution was drained and the glass-vessel with the resin was washed with two more portions of $CH_3OH$ ($2 \times 1$ mL). The combined methanolic solutions were added to the substance obtained from the TFA solution, the volatiles were removed *in vacuo* and the obtained product was dried thoroughly.

For characterization of nanocode **Sm-Tb-Eu** see the emission spectrum in Supplemetary Figure 22 und the $^{1}$H DOSY NMR studies in Section 1.6.

## 1.2. HPLC Traces

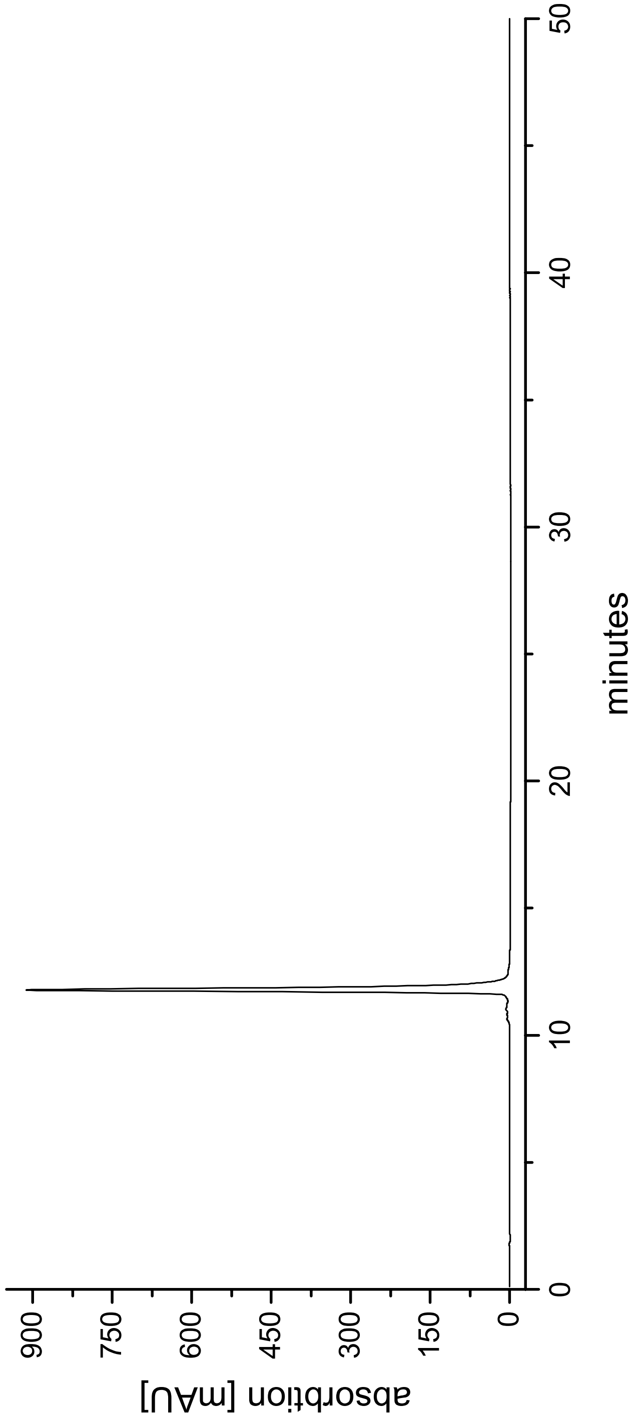


**Supplementary Figure 2.** Analytical HPLC trace of **S4-Sm** (HPLC program A).

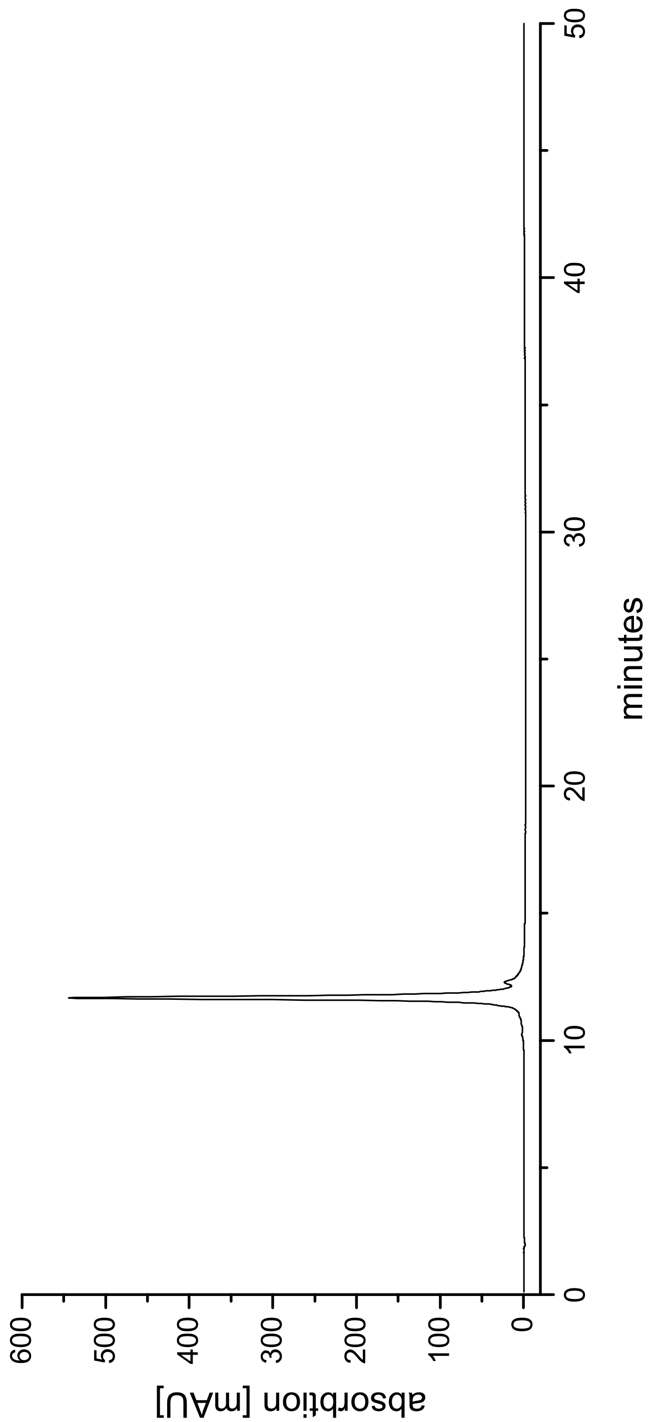


**Supplementary Figure 3.** Analytical HPLC trace of **S4-Eu** (HPLC program A).

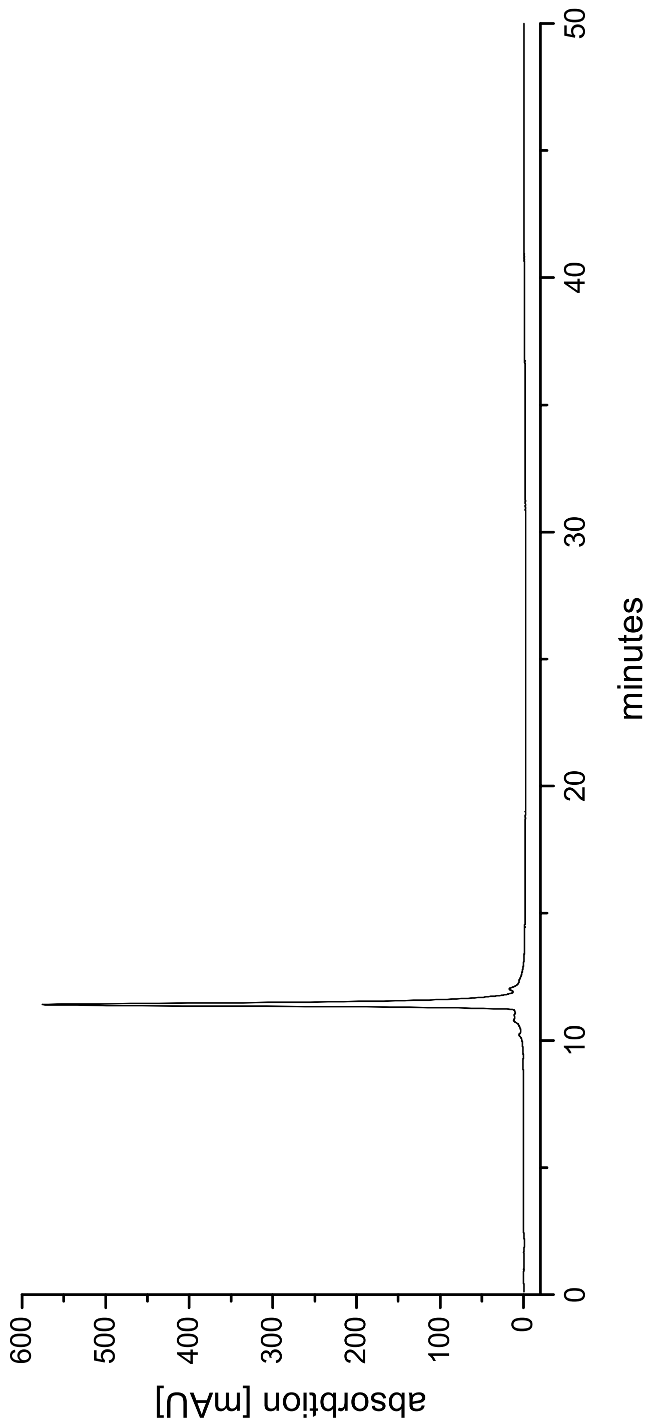


**Supplementary Figure 4.** Analytical HPLC trace of **S4-Tb** (HPLC program A).

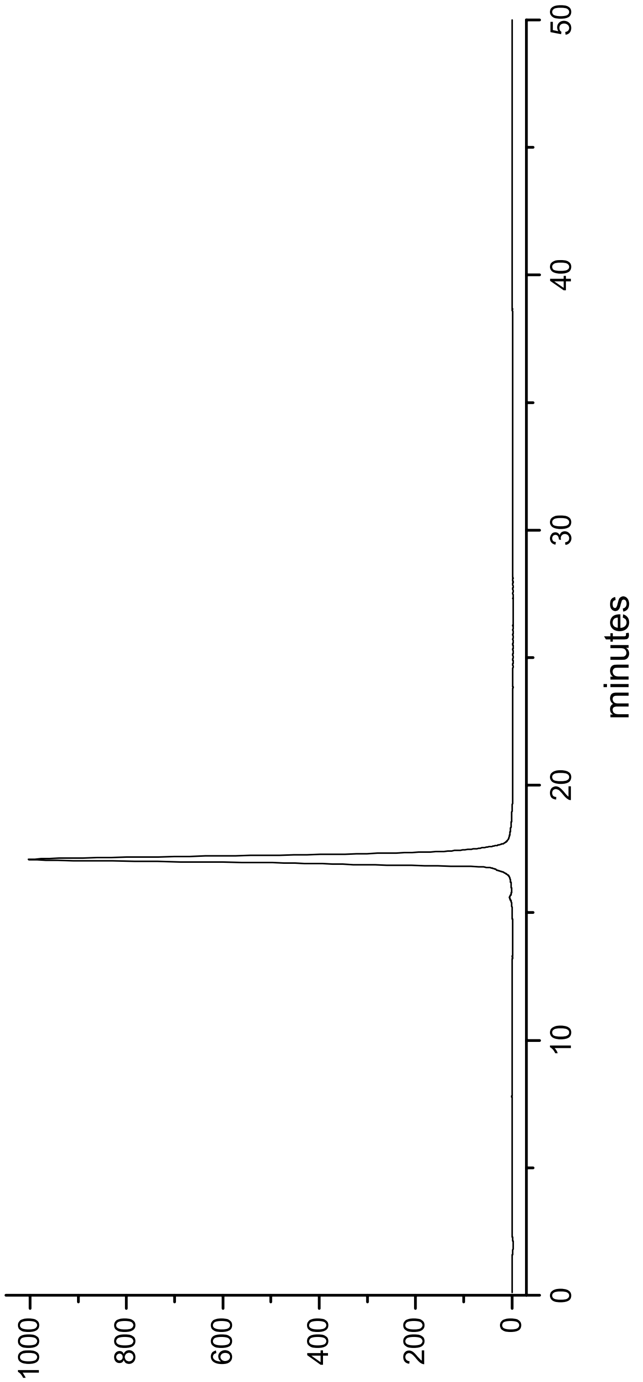


**Supplementary Figure 5.** Analytical HPLC trace of **Fmoc-Lys(Sm)** (HPLC program A).

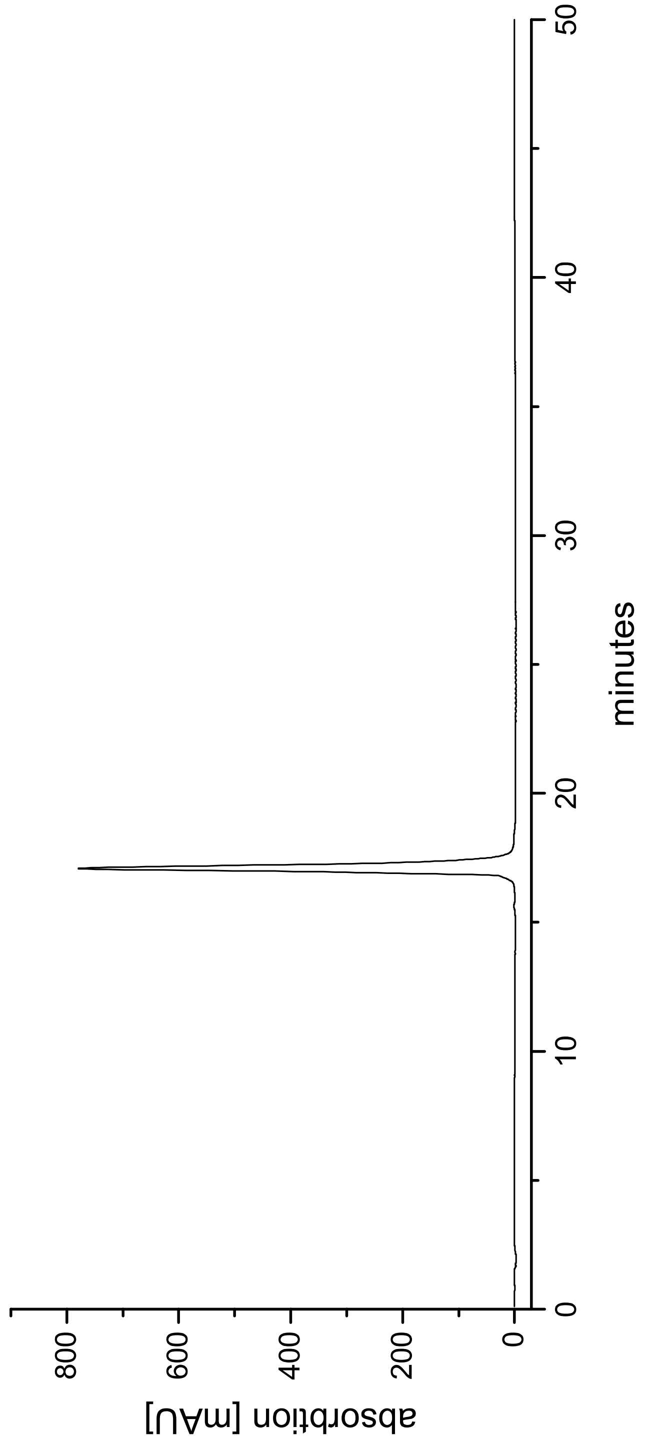


**Supplementary Figure 6.** Analytical HPLC trace of **Fmoc-Lys(Eu)** (HPLC program A).

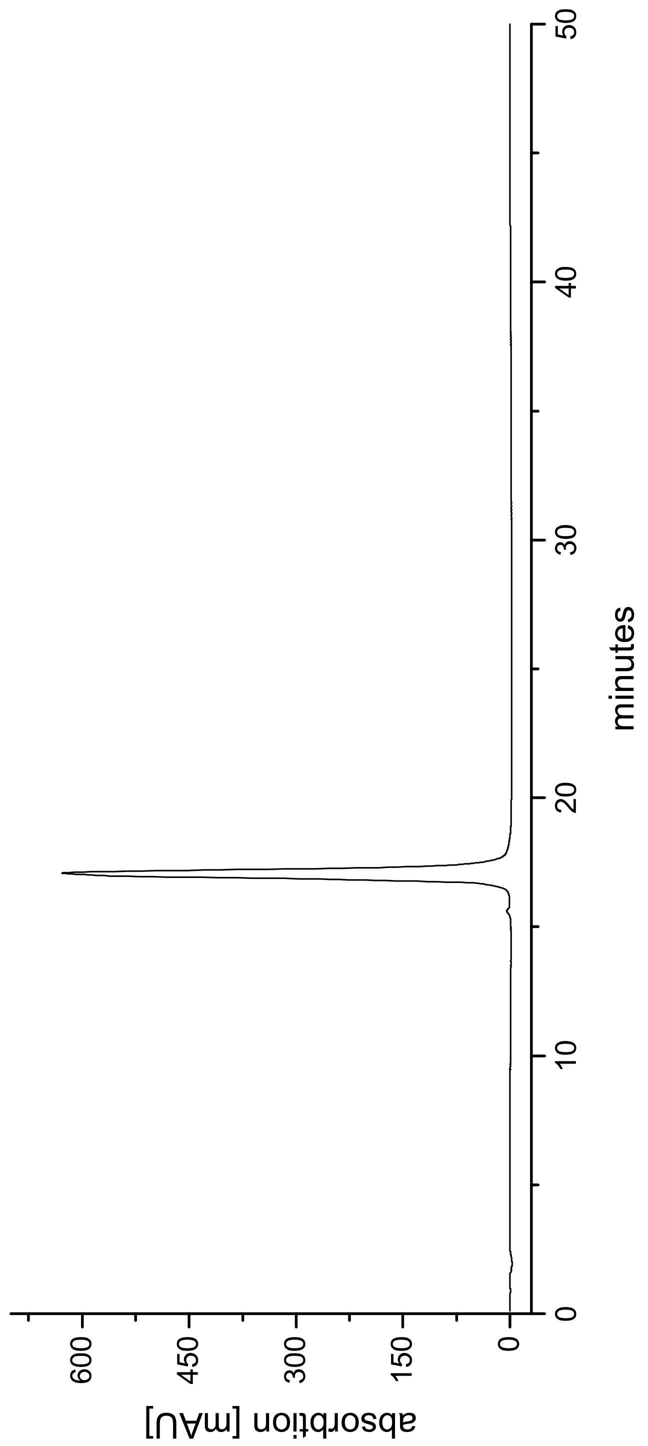


**Supplementary Figure 7.** Analytical HPLC trace of **Fmoc-Lys(Tb)** (HPLC program A).

## 1.3. $^{1}H$ NMR Spectra

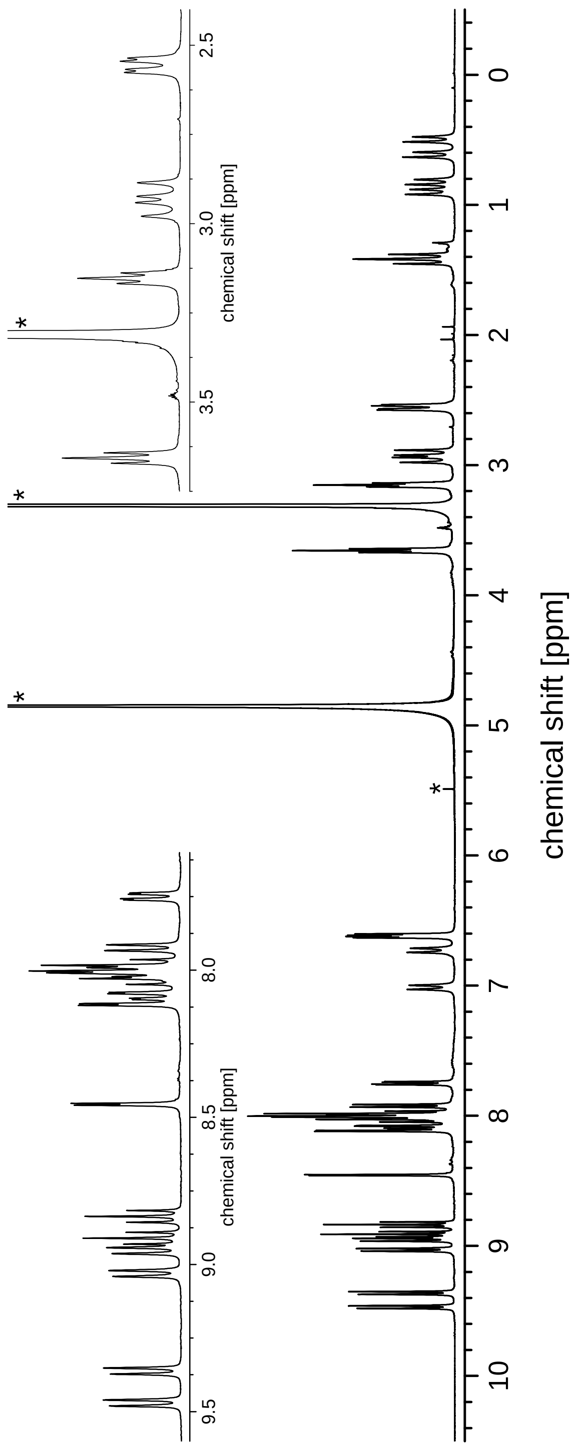


**Supplementary Figure 8.** $^{1}H$ NMR (400 MHz, $CD_3OD$) spectrum of **S4-Sm** (* residual solvent signals).

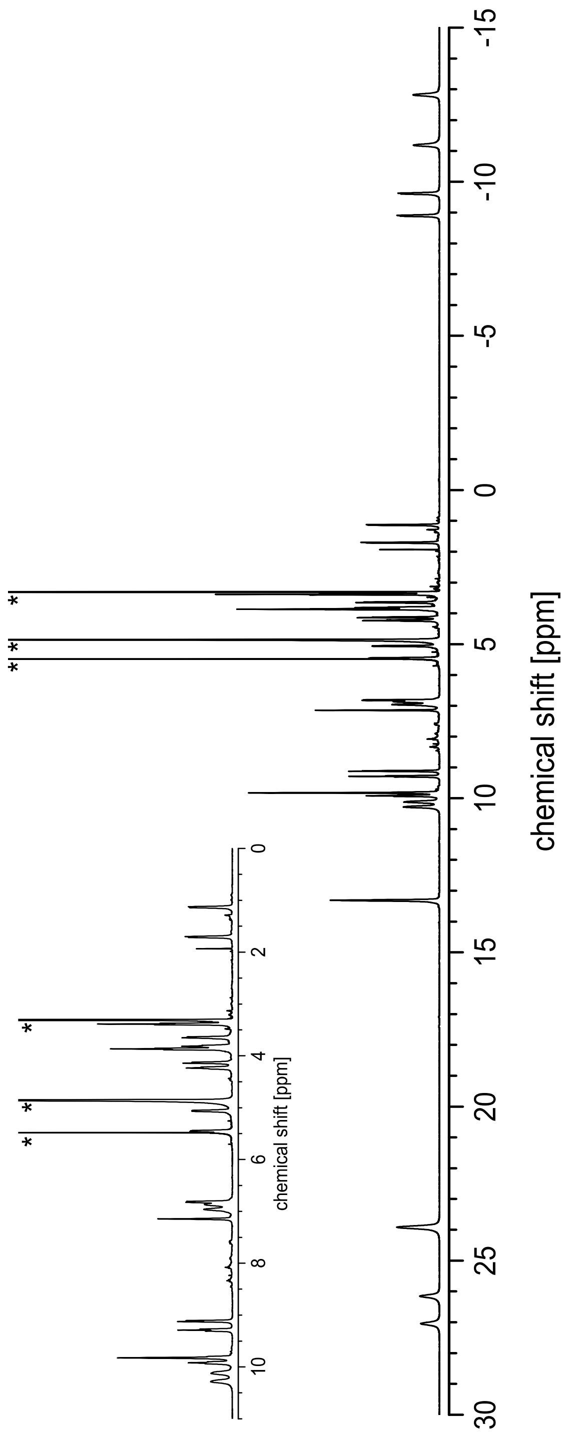


**Supplementary Figure 9.** $^{1}H$ NMR (400 MHz, $CD_3OD$) spectrum of **S4-Eu** (* residual solvent signals).

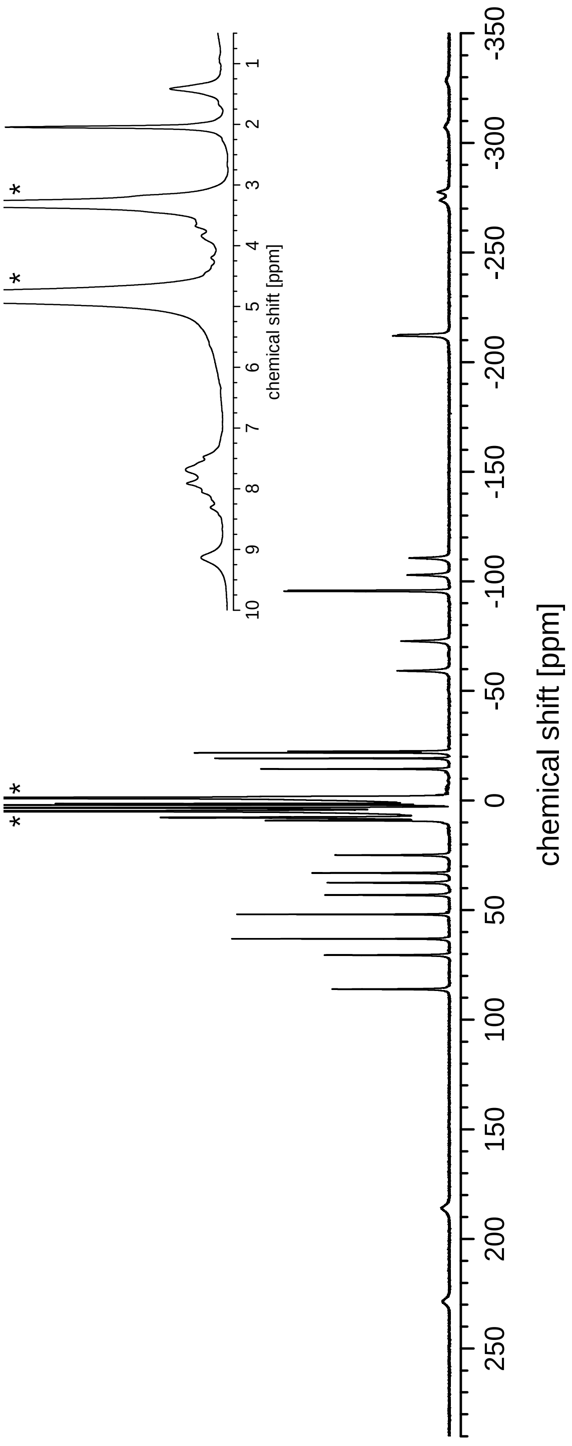


**Supplementary Figure 10.** $^{1}H$ NMR (500 MHz, $CD_3OD$) spectrum of **S4-Tb** (* residual solvent signals).

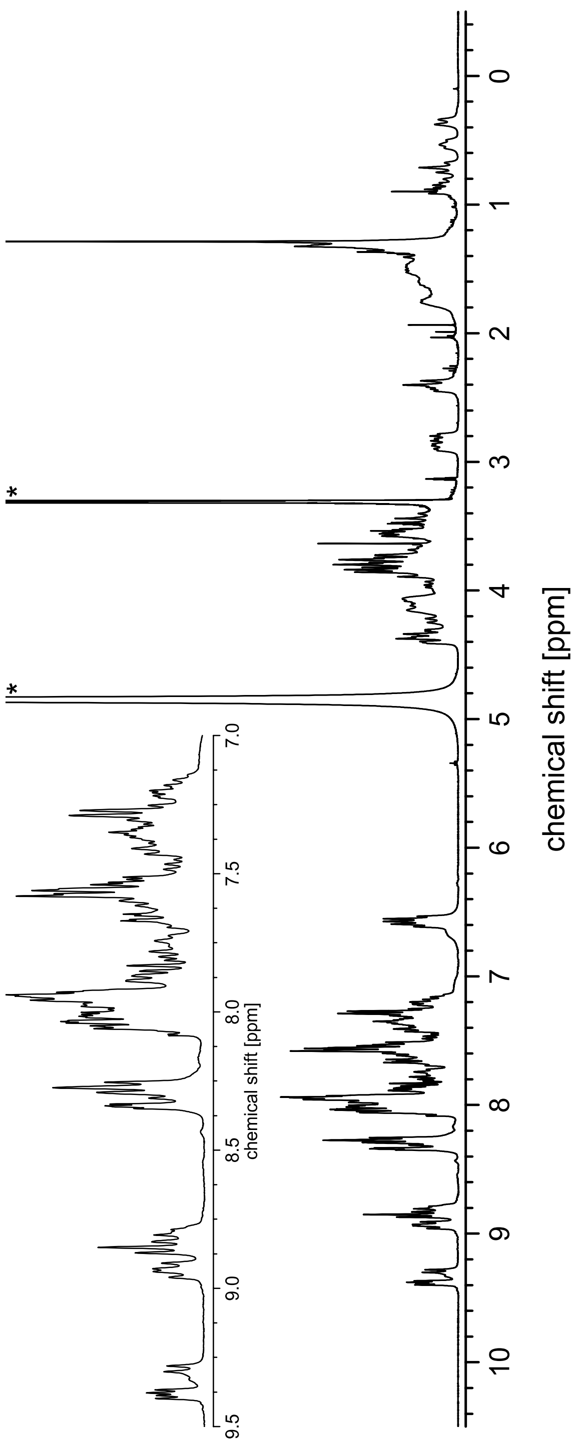


**Supplementary Figure 11.** $^{1}$H NMR (400 MHz, $CD_3OD$) spectrum of **Fmoc-Lys(Sm)** (* residual solvent signals).

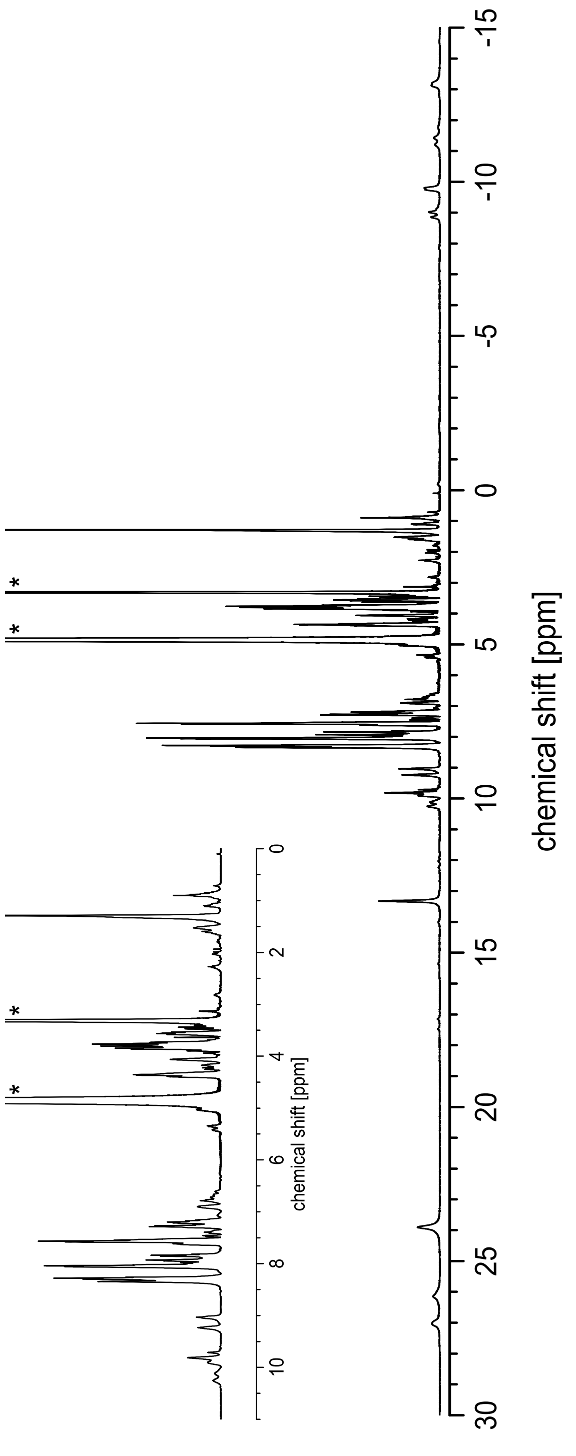


**Supplementary Figure 12.** $^{1}$H NMR (400 MHz, $CD_3OD$) spectrum of **Fmoc-Lys(Eu)** (* residual solvent signals).

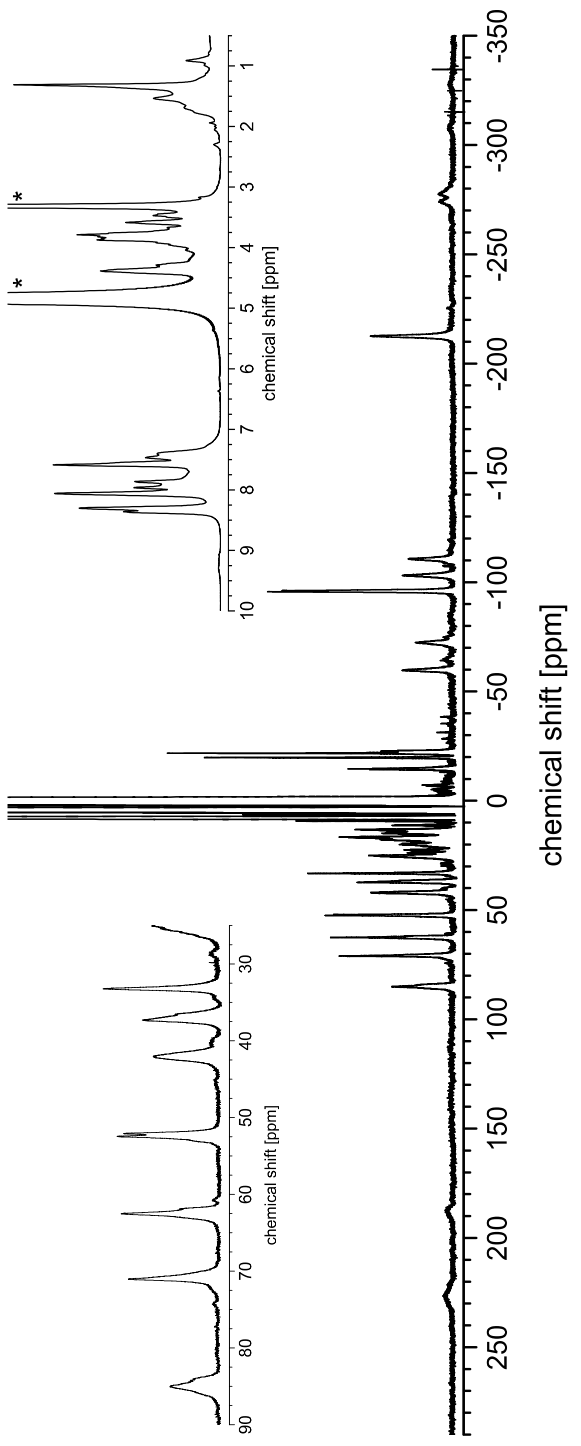


**Supplementary Figure 13.** $^{1}H$ NMR (400 MHz, $CD_3OD$) spectrum of **Fmoc-Lys(Tb)** (* residual solvent signals).

## 1.4. Absorption/Luminescence Spectra of 4-Ln

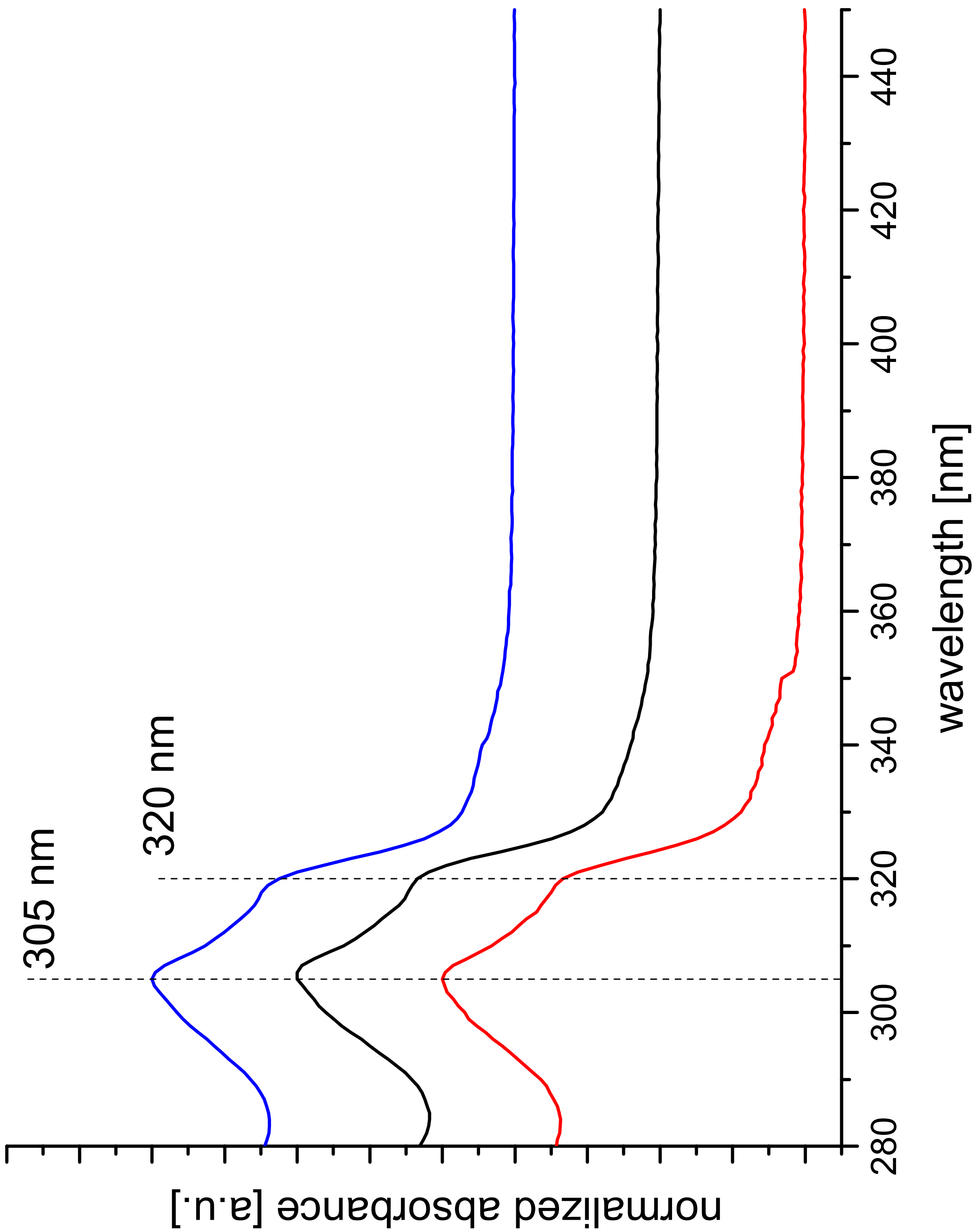


**Supplementary Figure 14.** UV/vis absorption spectra of **S4-Ln** in $CD_3OD$ (Sm: black; Eu: red; Tb: blue). For the sake of clarity the spectra are represented with an arbitrary horizontal offset.

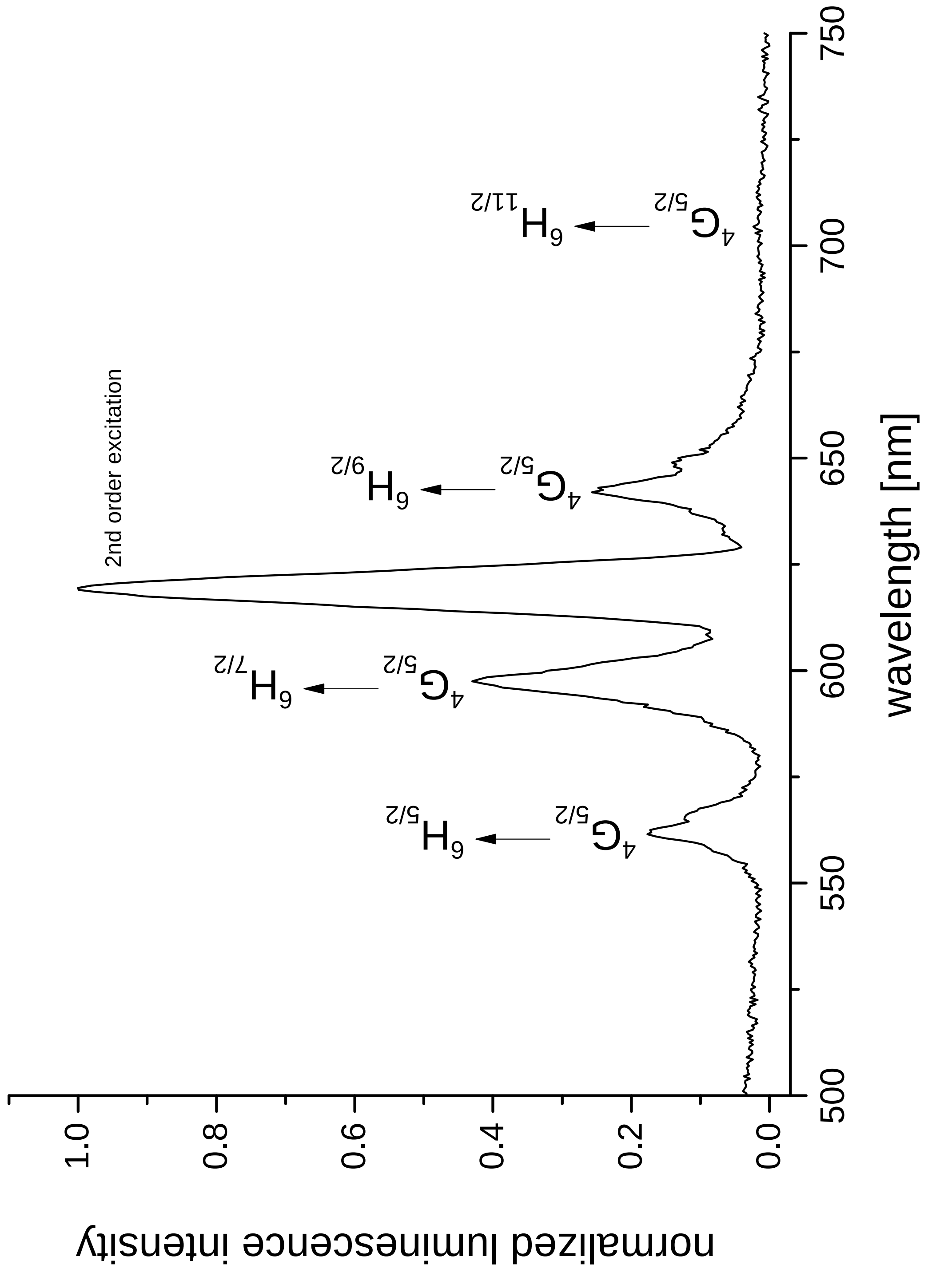


**Supplementary Figure 15.** Steady state emission spectrum of **S4-Sm** in $CD_3OD$ ($A_{310\ nm}$ = 0.15, $\lambda_{exc}$ = 310 nm).

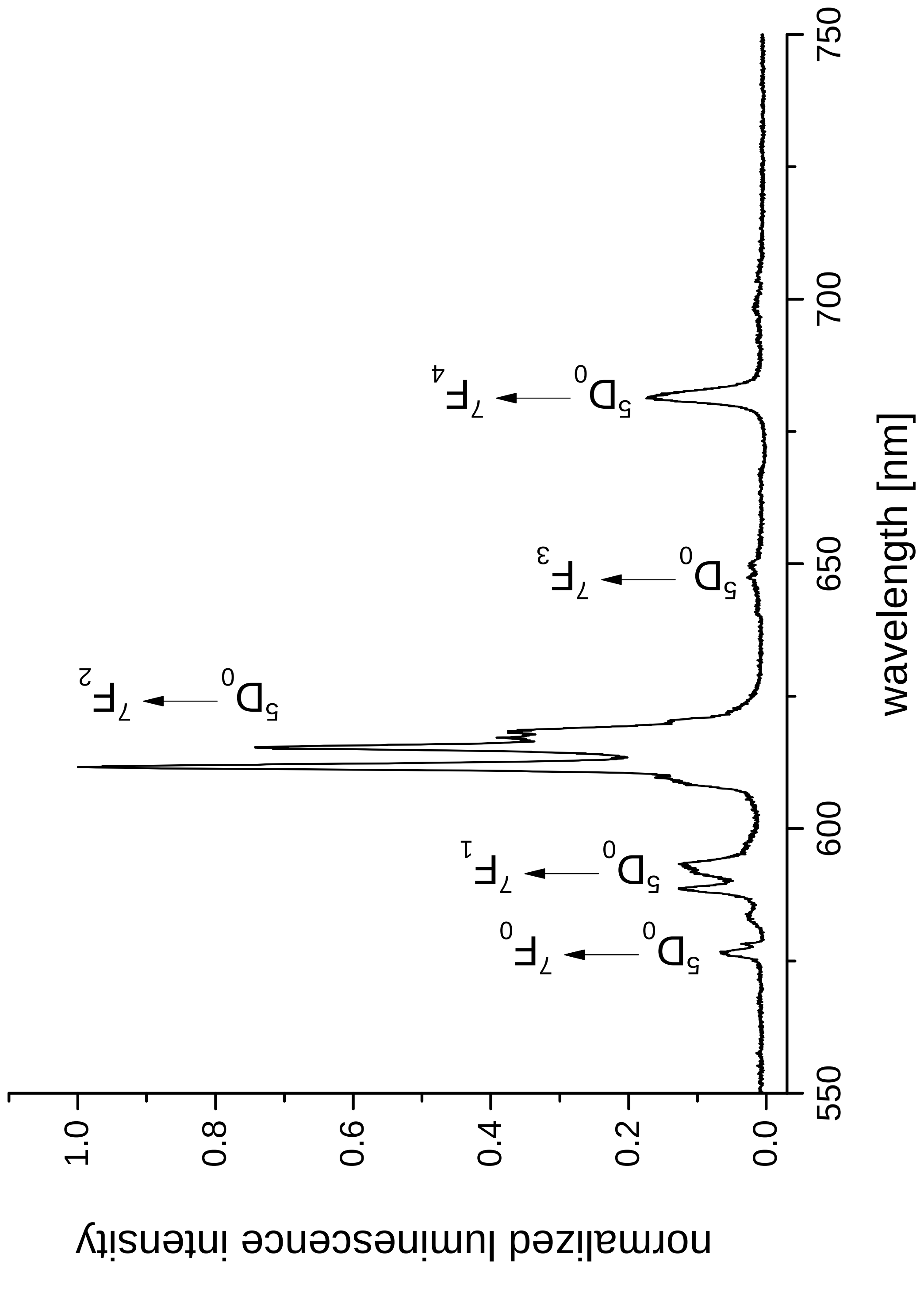


**Supplementary Figure 16.** Steady state emission spectrum of **S4-Eu** in $CD_3OD$ ($A_{320\ nm}$ = 0.05, $\lambda_{exc}$ = 320 nm).

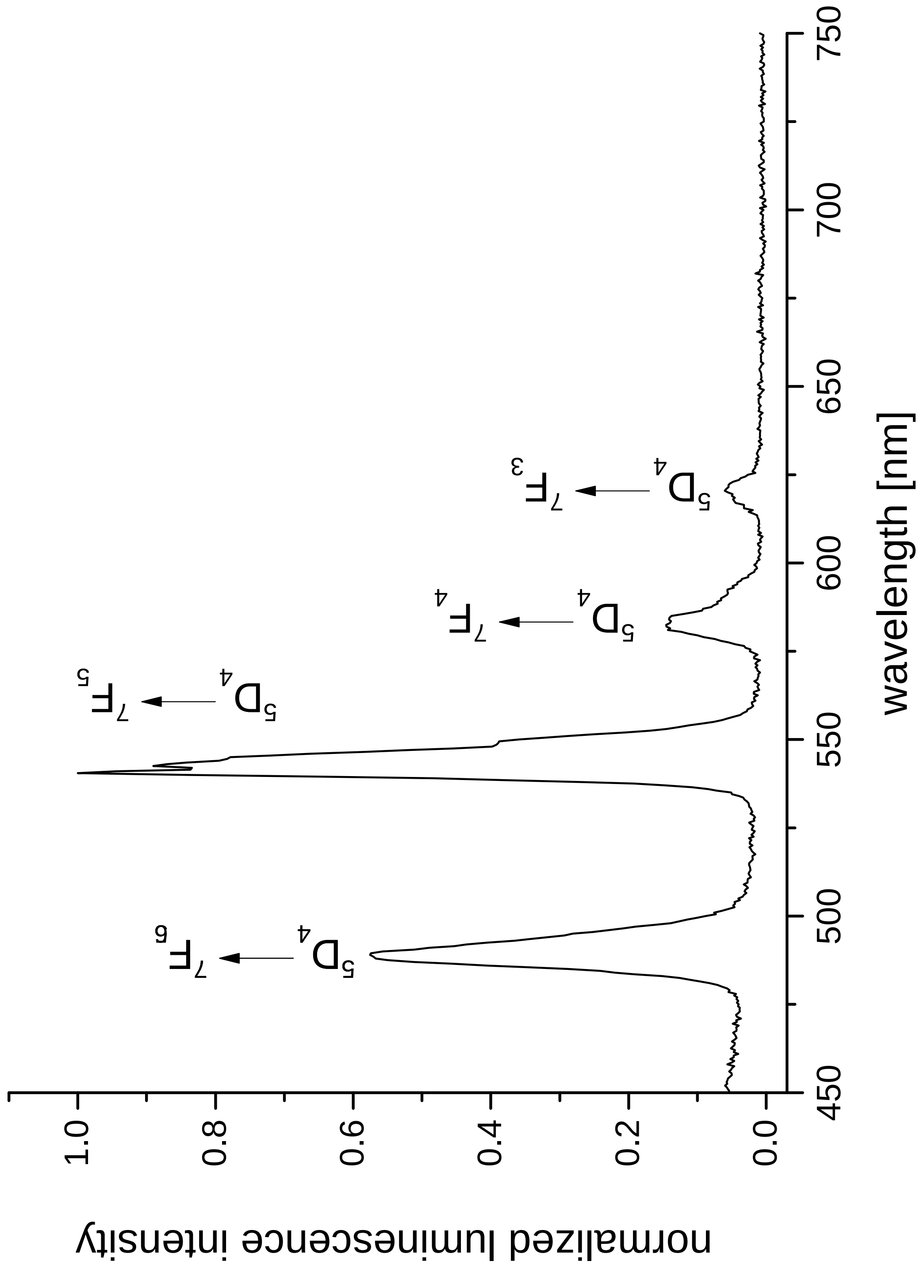


**Supplementary Figure 17.** Steady state emission spectrum of **S4-Tb** in $CD_3OD$ ($A_{305\ nm}$ = 0.20, $\lambda_{exc}$ = 305 nm, excitation path: long pass filter LP399).

## 1.5. Absorption/Luminescence Spectra of Fmoc-Lys(Ln) and Nanocode Sm-Tb-Eu

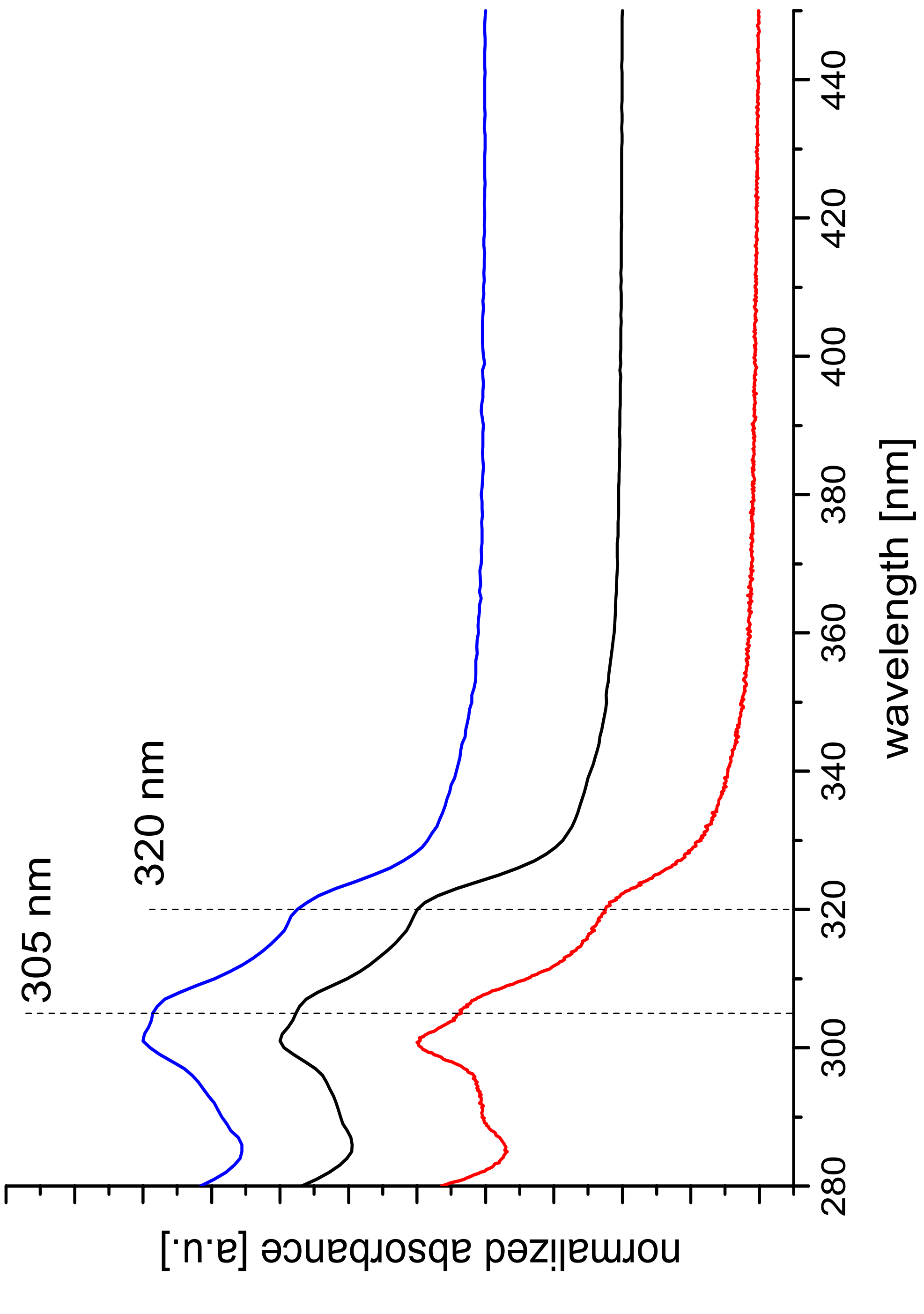


**Supplementary Figure 18.** UV-vis absorption spectrum of **Fmoc-Lys(Ln)** in $CD_3OD$ (Sm: black; Eu: red; Tb: blue). For the sake of clarity the spectra are represented with an arbitrary horizontal offset.

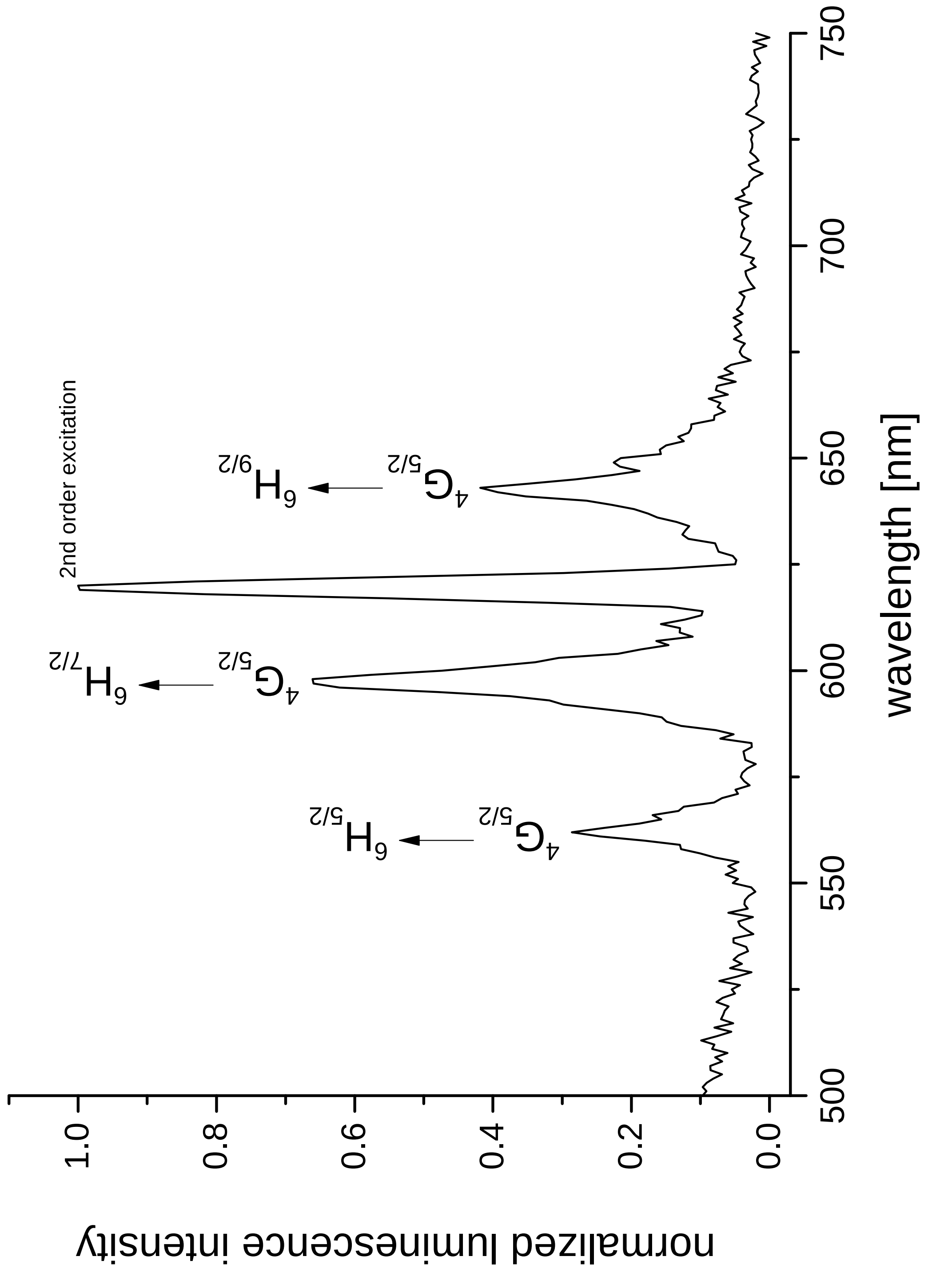


**Supplementary Figure 19.** Steady state emission spectrum of **Fmoc-Lys(Sm)** in $CD_3OD$ ($A_{310nm}$ = 0.24, $\lambda_{exc}$ = 310 nm).

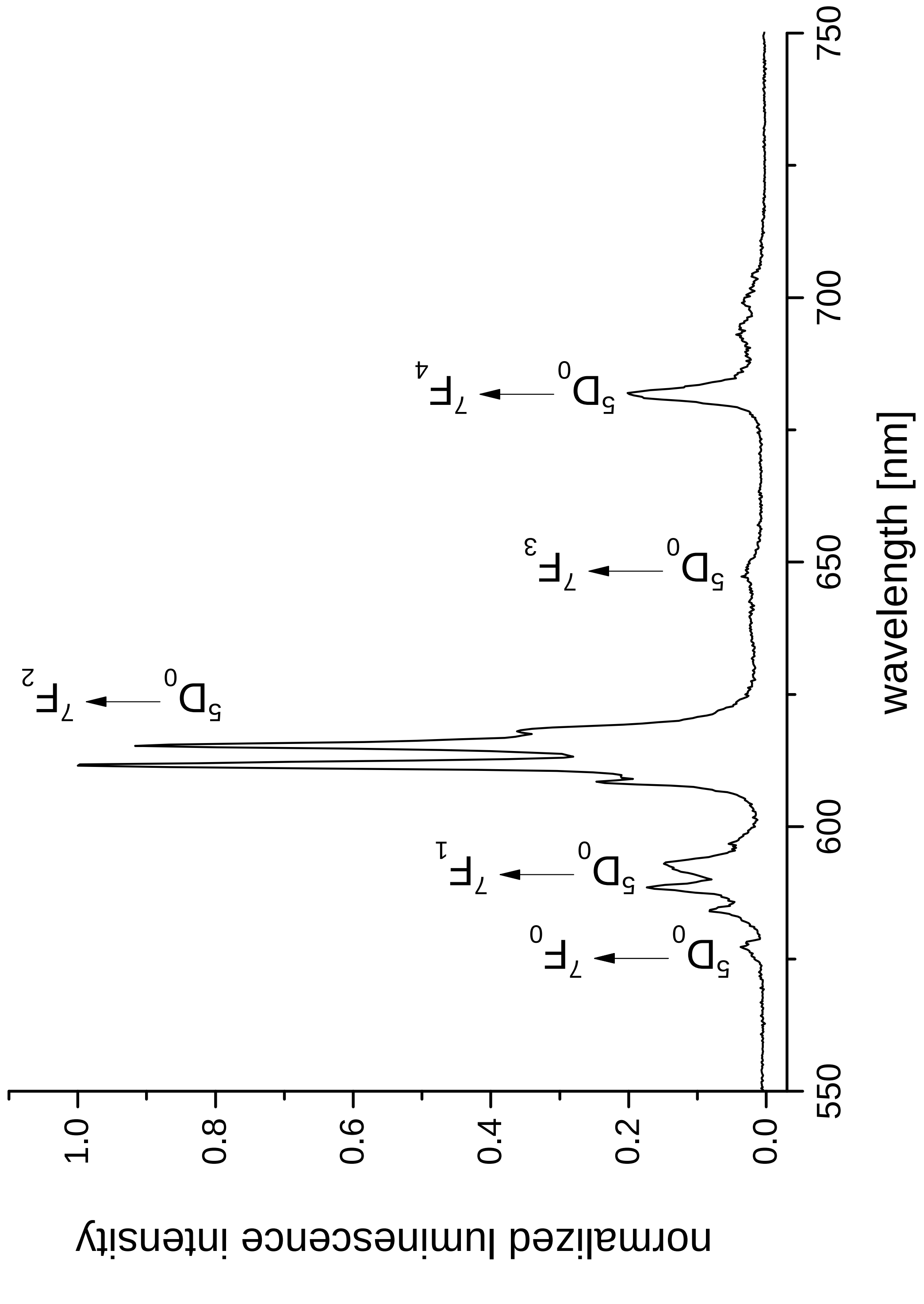


**Supplementary Figure 20.** Steady state emission spectrum of **Fmoc-Lys(Eu)** in $CD_3OD$ ($A_{320nm}$ = 0.03, $\lambda_{exc}$ = 320 nm).

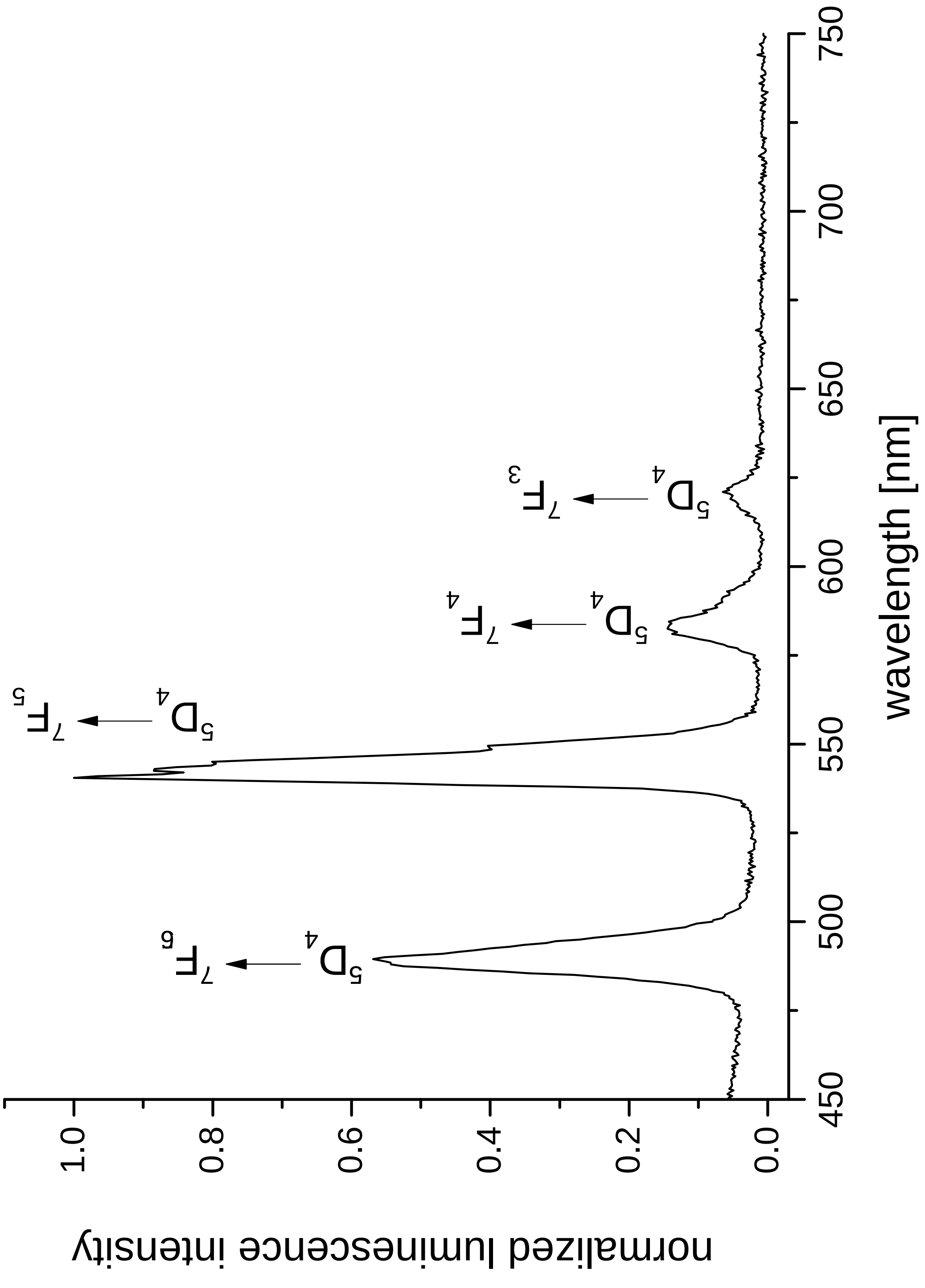


**Supplementary Figure 21.** Steady state emission spectrum of **Fmoc-Lys(Tb)** in $CD_3OD$ ($A_{305nm}$ = 0.12, $\lambda_{exc}$ = 305 nm, excitation path: long pass filter LP399).

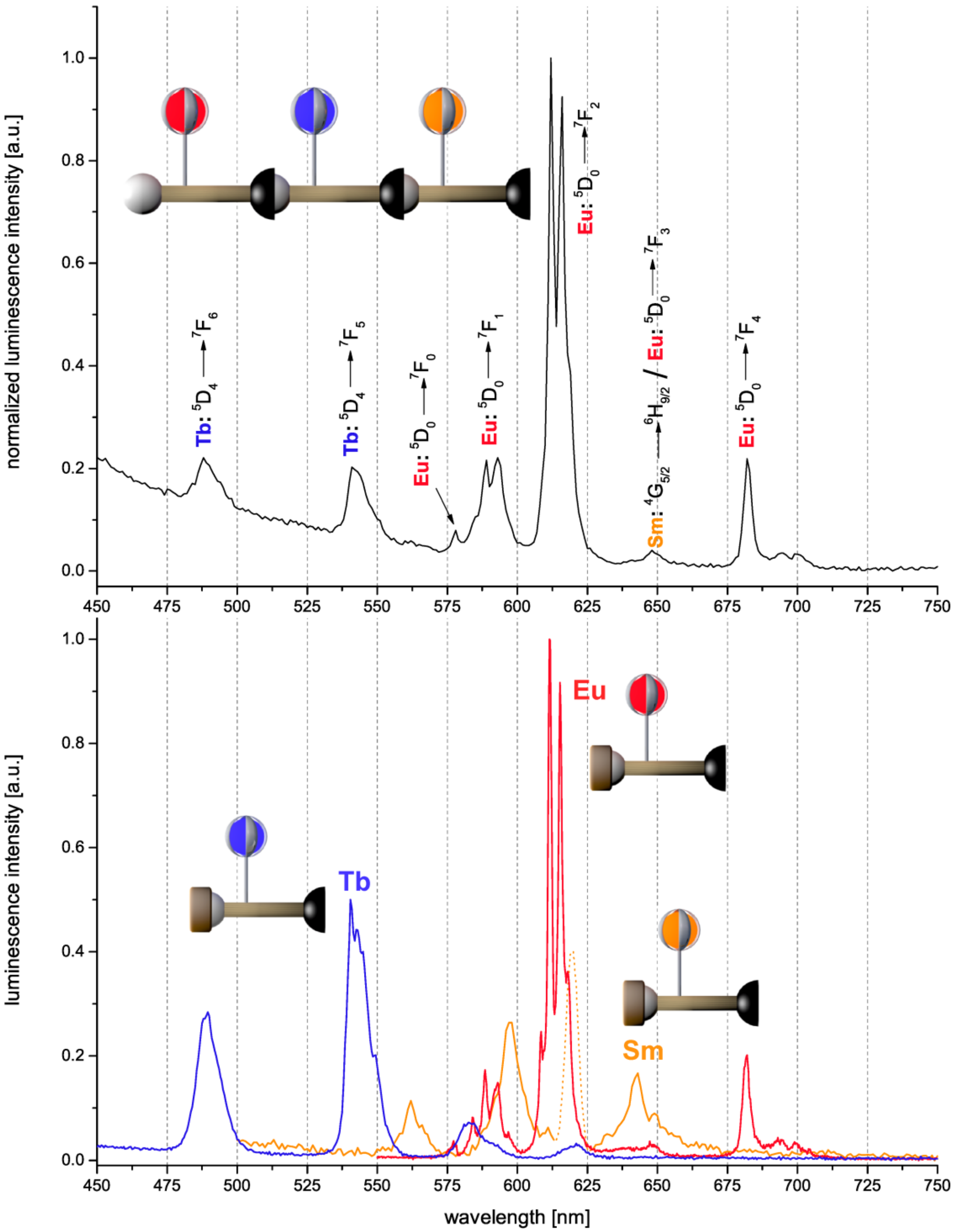


**Supplementary Figure 22.** Comparison of lanthanoid emission spectra: Top – Nanocode **Sm-Tb-Eu** ($CD_3OD$; $\lambda_{exc}$ = 320 nm, emission path: long pass filter LP399); Bottom: Superimposed spectra for the three monomers **Fmoc-Lys(Ln)** ($CD_3OD$; Ln = Sm: $\lambda_{exc}$ = 310 nm; Ln = Tb: $\lambda_{exc}$ = 305 nm with long pass filter LP399 in the emission path; Ln = Eu: $\lambda_{exc}$ = 320 nm; dotted line: second order excitation peak).

## 1.6. $^{1}$H DOSY NMR Studies

All DOSY experiments were performed at 298 K on a Bruker Avance III HDX 700 NMR spectrometer ($^{1}$H: 700 MHz) using the pulse sequence ledbpgp2s and gaussian shaped gradient forms. The parameters p30 and D20 in the pulse sequence were determined for each sample individually. The maximum z-gradient strength were obtained from measuring a sample of 1 wt% $H_2O$ and 0.1 wt% $CuSO_4$ in $D_2O$, for which the diffusion coefficient for $D_2O$ was used with $D = 1.872 \cdot 10^{-9}$ m$^2$ s$^{-1}$.[3] The viscosity of each sample was calculated from the diffusion coefficients $D$ of the residual protonated solvents using Supplementary Equation 1 (vide infra). All samples were prepared in $CD_3OD$ with mass concentrations of ca. 1 mg mL$^{-1}$. The $^{1}$H DOSY NMR spectrum (Supplementary Figure 23) for nanocode **Sm-Tb-Eu** shows intense signals in the range below 6 ppm caused by the protons of solvent molecules and impurities (such as polyethylene glycol fragments which originate from the peptide synthesis resin) inhibiting an unambiguous identification of the signals of **Sm-Tb-Eu** in this spectral region. For the study of **Sm-Tb-Eu** we therefore focused on the aromatic signals between 6.5 ppm and 8.5 ppm which are unique and characteristic for **Sm-Tb-Eu** in this system.

Our model for the analysis of the DOSY data assumes the diffusion of hard spheres with hydrodynamic radius $r$, volume $V$, molar mass $M$, and effective density $\rho_{\mathrm{eff}}$. The Stokes-Einstein relation[4] together with Supplementary Equations 2 and 3 yields Supplementary Equation 4:

$$D = \frac{k_{\mathrm{B}} \cdot T}{6 \cdot \pi \cdot \eta \cdot r} \qquad (1)$$

(with $k_{\mathrm{B}}$: Boltzmann's constant, $T$: absolute temperature, $\eta$: viscosity)

$$V = \tfrac{4}{3}\pi \cdot r^3 \qquad (2)$$

$$V = \frac{M}{\rho_{\mathrm{eff}} \cdot N_{\mathrm{A}}} \qquad (3)$$

(with $N_{\mathrm{A}}$: Avogardo's constant)

$$D = \frac{k_{\mathrm{B}} \cdot T}{6 \cdot \pi \cdot \sqrt[3]{\frac{3}{4\pi \cdot \rho_{\mathrm{eff}} \cdot N_{\mathrm{A}}}}} \cdot \frac{1}{\eta} \cdot \frac{1}{\sqrt[3]{M}} \qquad (4)$$

The first term in Supplementary Equation 4 is constant at a fixed temperature and assuming constant effective density $\rho_{\mathrm{eff}}$ for all compounds. Especially the latter assumption is not universally valid (e.g. when analyzing data for organic compounds without heavy atoms and

lanthanoid complexes) but has been shown to work quite well when comparing only similar species (e.g. proteins, polymers, etc.).[5,6] Therefore, the following proportionality holds in our case (Supplementary Equation 5):

$$D \cdot \eta \propto \frac{1}{\sqrt[3]{M}} \qquad (5)$$

Due to the crucial importance of the similarity of the chemical species for the validity of the analysis (vide supra), we only chose well-defined, non-aggregating lanthanoid complexes of various sizes as reference compounds for the analysis of the nanocode **Sm-Tb-Eu**. Supplementary Figure 25 shows the structures and molar masses associated with the species analyzed. The corresponding data for the DOSY measurements are summarized in Supplementary Table 2. The plot $D \cdot \eta$ versus $M^{-1/3}$ according to Supplementary Equation 5 is shown in Supplementary Figure 23, which shows the expected good linear fit. This is very good confirmation of the expected molar mass of nanocode **Sm-Tb-Eu**.

**Supplementary Table 2.** $^1$H NMR DOSY data for **Sm-Tb-Eu** and the reference compounds (Supplementary Figure 25).

| | $[Sm(bpy2a)]^-$ (ref. 7) | $[Lu([D_4]\text{-}bpy_3O_2)(CF_3COO)]^{2+}$ (ref. 8) | $[\{L^{Me}Sm(O^iPr)\}_2]$ (ref. 9) | **Sm-Tb-Eu** (this work) |
|---|---|---|---|---|
| $M^a$ [g mol$^{-1}$] | 634.74 | 898.70 | 1232.04$^b$ | 3578.49 |
| $\frac{1}{\sqrt[3]{\frac{M}{(g\ mol^{-1})}}}$ | 0.116 | 0.104 | 0.0933$^b$ | 0.0654 |
| $D$ [$10^{-10}$ m$^2$ s$^{-1}$] | 7.06 | 5.82 | 5.89$^b$ | 4.12 |
| p$D$ = -lg($D$/(m$^2$ s$^{-1}$)) | 9.15 | 9.24 | 9.23$^b$ | 9.38 |
| $\eta$ [mPa·s] | 0.58$^c$ ($CD_3OD$) | 0.64$^c$ ($CD_3OD$) | 0.54$^d$ ($CDCl_3$) | 0.61$^c$ ($CD_3OD$) |
| $D \cdot \eta$ [$10^{-12}$ N] | 410 | 372 | 318$^b$ | 251 |

$^a$ See Supplementary Figure 25 for structural details; $^b$ Taken from ref. 9; $^c$ Calculated for each individual sample from tabulated viscosity and self-diffusion data for the solvents, see ref. 3; $^d$ Literature value from ref. 5.

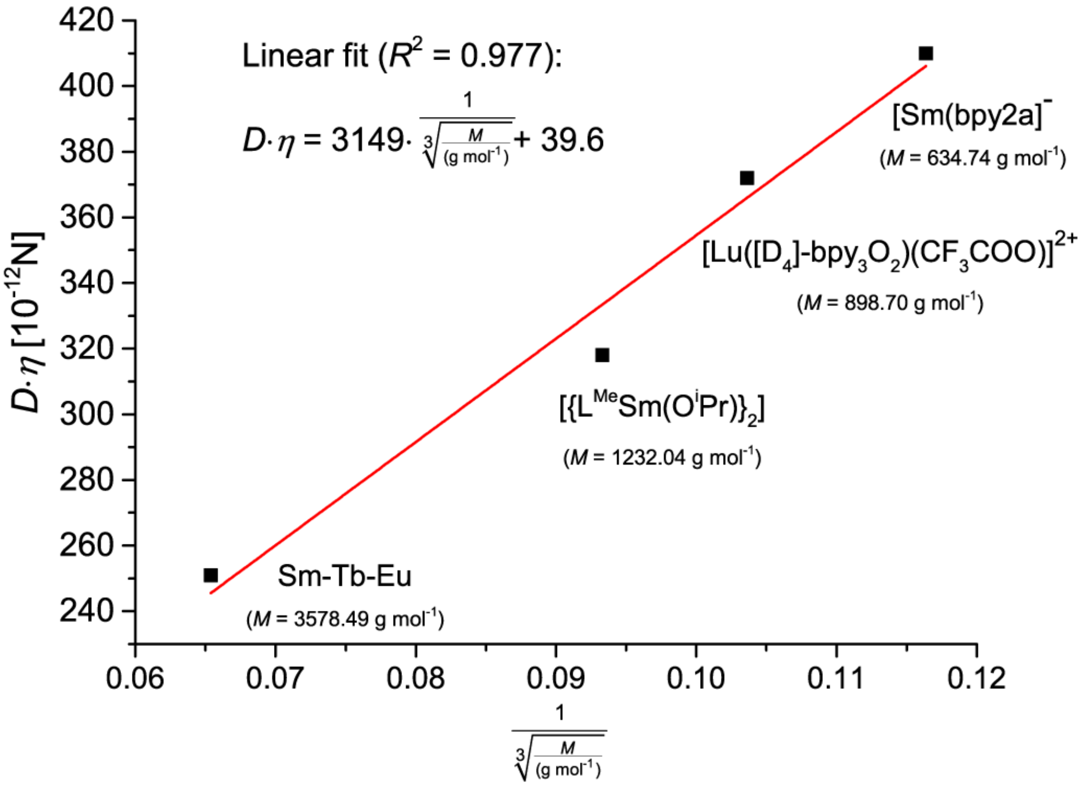


**Supplementary Figure 23.** Plot of the product of the experimentally determined diffusion coefficients *D* and the viscosity $\eta$ of the solvents versus $M^{-1/3}$ of the compounds studied via $^1$H DOSY NMR experiments (see Supplementary Figure 25 for compounds and Supplementary Table 2 for underlying data).

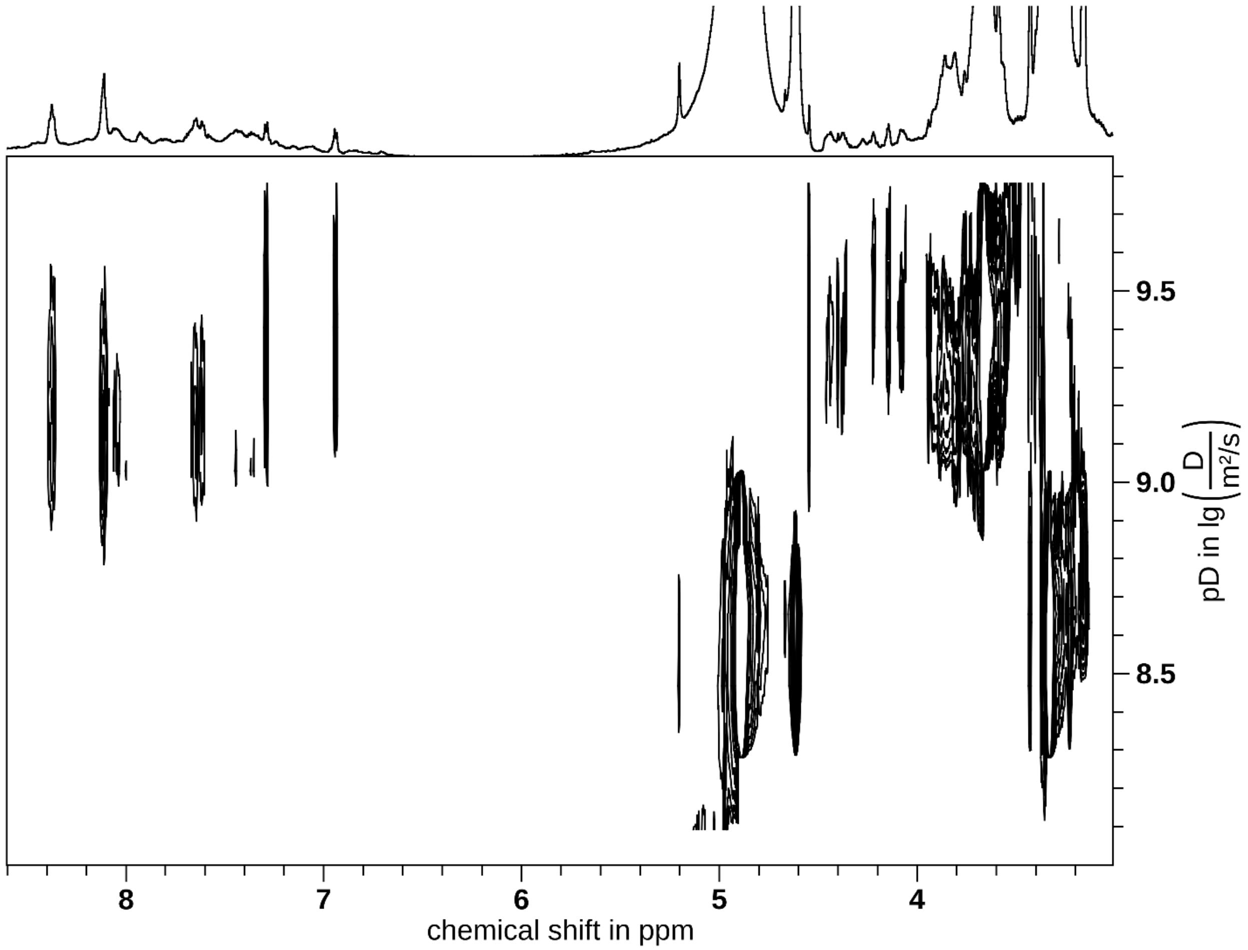


**Supplementary Figure 24.** $^1$H DOSY NMR spectrum ($CD_3OD$, 700 MHz) of the nanocode **Sm-Tb-Eu**.

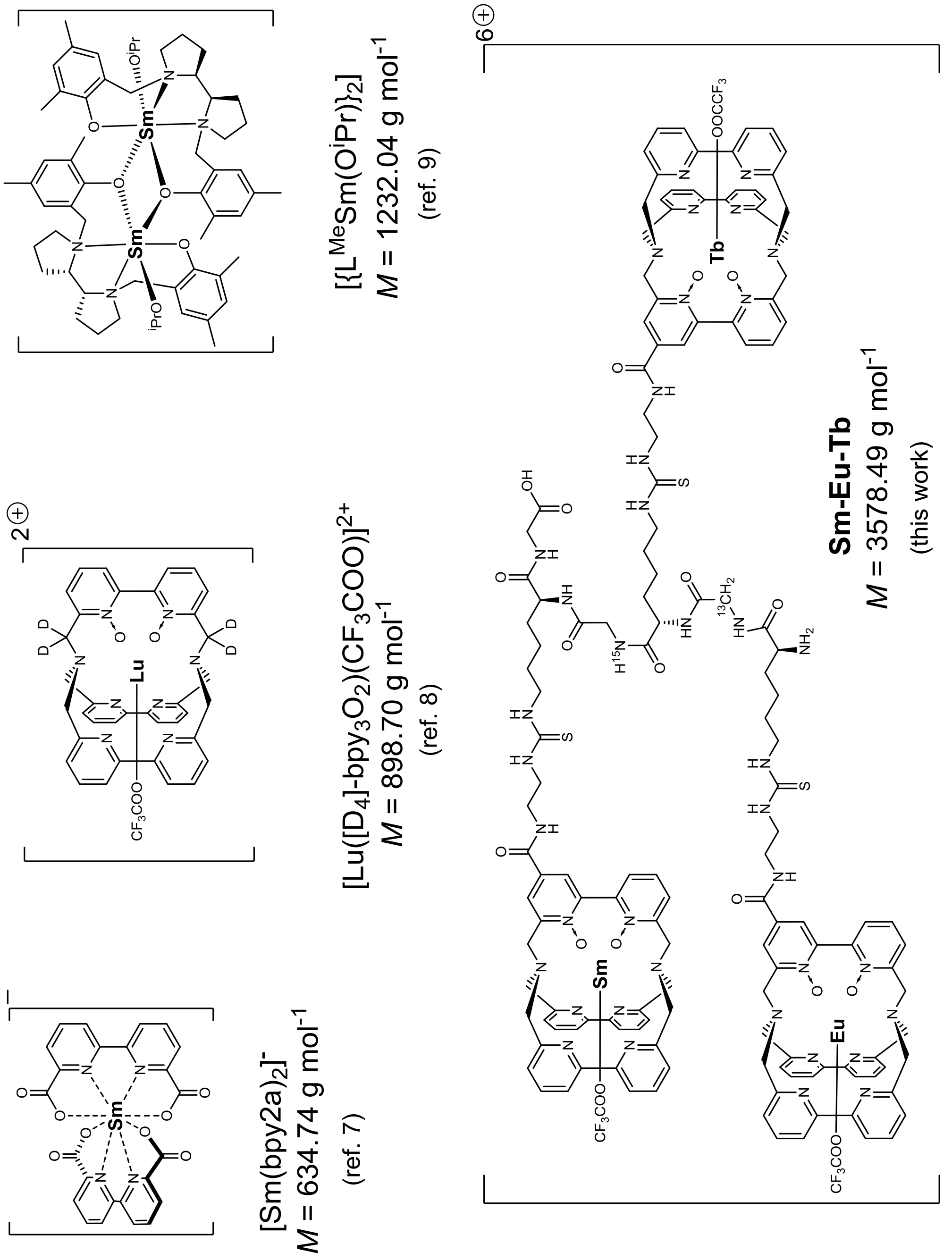


**Supplementary Figure 25.** Structures of the compounds used for the $^1$H DOSY NMR analysis (see Supplementary Table 2) with corresponding molar masses *M*.

## 2. Supplementary References


1. Kreidt, E., Bischof, C., Platas-Iglesias, C. & Seitz, M. Magnetic anisotropy in functionalized bipyridyl cryptates. *Inorg. Chem.* **55**, 5549-5557 (2016).

2. Chan, W. & White, P. *Fmoc Solid Phase Peptide Synthesis* (Oxford University Press, Oxford, 2000).

3. Bruker Corporation, *Almanac 2014* (Bruker Corporation, Rheinstetten, 2014); URL: www.bruker.com/almanac

4. Einstein, A. Über die von der molekularkinetischen Theorie der Wärme geforderte Bewegung von in ruhenden Flüssigkeiten suspendierten Teilchen. *Ann. Phys.* **322**, 549-560 (1905).

5. Evans, R., Dal Poggetto, G., Nilsson, M. & Morris, G. A. Improving the interpretation of small molecule diffusion coefficients. *Anal. Chem.* **90**, 3987-3994 (2018).

6. Poh, A. W. J., Aguilar, J. A., Kenwright, A. M., Mason, K. & Parker, D. Aggregation of rare earth coordination complexes in solution studied by paramagnetic and DOSY NMR. *Chem. Eur. J.* **24**, 16170-16175 (2018).

7. Wahsner, J. & Seitz, M. Non-radiative deactivation of lanthanoid excited states by inner-sphere carboxylates. *Inorg. Chem*. **54**, 10841-10848 (2015).

8. Doffek, C. et al. Understanding the quenching effects of aromatic C-H- and C-D-oscillators in near-IR lanthanoid luminescence. *J. Am. Chem. Soc.* **134**, 16413-16423 (2012).

9. Beament, J., Kociok-Köhn, G., Jones, M. D. & Buchard, A. Bipyrrolidine salan alkoxide complexes of lanthanides: Synthesis, characterisation, activity in the polymerisation of lactide and mechanistic investigation by DOSY NMR. *Dalton Trans.* ***47,*** 9164-9172 (2018).
